# Low-dose AAV-CRISPR-mediated liver-specific knock-in restored hemostasis in neonatal hemophilia B mice with subtle antibody response

**DOI:** 10.1038/s41467-022-34898-y

**Published:** 2022-11-25

**Authors:** Xiangjun He, Zhenjie Zhang, Junyi Xue, Yaofeng Wang, Siqi Zhang, Junkang Wei, Chenzi Zhang, Jue Wang, Brian Anugerah Urip, Chun Christopher Ngan, Junjiang Sun, Yuefeng Li, Zhiqian Lu, Hui Zhao, Duanqing Pei, Chi-Kong Li, Bo Feng

**Affiliations:** 1 School of Biomedical Sciences, MOE Key Lab, Faculty of Medicine; Institute for Tissue Engineering and Regenerative Medicine (iTERM), The Chinese University of Hong Kong, Hong Kong SAR, China; 2 Centre for Regenerative Medicine and Health, Hong Kong Institute of Science & Innovation, Chinese Academy of Sciences, Hong Kong SAR, China; 3 Guangzhou Institute of Biomedicine and Health, Chinese Academy of Sciences, Guangzhou, 510530 China; 4 Division of Molecular Pharmaceutics, Eshelman School of Pharmacy, University of North Carolina, Chapel Hill, NC USA; 5 Guangdong Landau Biotechnology Co.Ltd, Guangzhou, 510555 China; 6 Department of Cardiothoracic Surgery, Shanghai Jiao Tong University Affiliated Sixth People’s Hospital, Shanghai, 200233 China; 7 The Chinese University of Hong Kong, Shenzhen Research Institute, Shenzhen, 518000 China; 8 Laboratory of Cell Fate Control, School of Life Sciences, Westlake University, Hangzhou, 310024 China; 9 Department of Pediatrics, Hong Kong Children’s Hospital, The Chinese University of Hong Kong, Hong Kong SAR, China

**Keywords:** Targeted gene repair, Molecular medicine

## Abstract

AAV-delivered CRISPR/Cas9 (AAV-CRISPR) has shown promising potentials in preclinical models to efficiently insert therapeutic gene sequences in somatic tissues. However, the AAV input doses required were prohibitively high and posed serious risk of toxicity. Here, we performed AAV-CRISPR mediated homology-independent knock-in at a new target site in *mAlb* 3’UTR and demonstrated that single dose of AAVs enabled long-term integration and expression of *hF9* transgene in both adult and neonatal hemophilia B mice (*mF9 −/−)*, yielding high levels of circulating human Factor IX (hFIX) and stable hemostasis restoration during entire 48-week observation period. Furthermore, we achieved hemostasis correction with a significantly lower AAV dose (2 × 10^9^ vg/neonate and 1 × 10^10^ vg/adult mouse) through liver-specific gene knock-in using hyperactive *hF9*^*R338L*^ variant. The plasma antibodies against Cas9 and AAV in the neonatal mice receiving low-dose AAV-CRISPR were negligible, which lent support to the development of AAV-CRISPR mediated somatic knock-in for treating inherited diseases.

## Abstract (web summary)

Here, the authors develop an AAV-CRISPR mediated somatic knock-in. They apply this system to restore hemostasis in neonatal hemophilia B mice and show liver-specificity of the knock-in and low serum antibody production.

## Introduction

Recombinant adeno-associated virus (AAV)^1^ coupled with CRISPR technologies^2,3^ provide tremendous potential in developing therapeutic approaches to permanently reverse disease-causing genetic defects^4,5^. Targeted insertion of a normal sequence to restore the gene function offers broad therapeutic potential regardless of mutation type and has been an attractive strategy. To establish a proof of concept, inherited hemophilia B caused by *F9* gene mutations has been intensively used as a disease model^6^. Through the homology-directed repair (HDR) mechanism, human *F9* gene exons (*hF9* Ex2–8) have been knocked-in in mice using AAV-delivered zinc finger nuclease (ZFN)^7,8^, while knock-in of *mF9* Ex2-8^9^ and hyperactive *hF9*^*R338L*^ variant (hFIX-Padua)^10^ were achieved using AAV-delivered CRISPR/SaCas9.

Recently, a homology-independent knock-in strategy was developed by exploiting the non-homologous end-joining (NHEJ) DNA repair mechanism, which demonstrated superior efficiency of targeted insertion in zebrafish and mammalian cells compared to the HDR-based approach^11,12^. In applying AAV-CRISPR to explore NHEJ knock-in in vivo, Suzuki *et al*. reported the restoration of *Mertk* gene function in rat retina^13^, while Zhang et al. and Chen et al. successfully rescued *mF8* gene defects in hemophilia A mice^14,15^. As homologous sequences are not required, NHEJ knock-in provides greater capacity and flexibility for AAV-based donor sequence delivery when performing in vivo gene editing.

To date, the therapeutic efficacy of AAV-mediated in vivo knock-in, via either HDR- or NHEJ-based strategies, has been broadly confirmed in preclinical models^16,17^. However, effective in vivo knock-in was mostly achieved at the expense of high-dose AAV inputs, which is associated with significant safety risks and hefty production costs for clinical adoption.

In this study, we systematically investigated the AAV-CRISPR mediated in vivo NHEJ knock-in and showed that a careful selection of targeting reagents and strategies can support effective gene knock-in with a much lower and safer AAV input. Through targeting at the proximal *mAlb* 3′UTR, high levels of liver-specific transgene expression were induced. Single-dose AAV administration in both adult and neonatal mice yielded robust *hF9* knock-in, which sustained hFIX production and corrected hemostasis in hemophilia B mice throughout the 48-week observation period. With liver-specific genome editing and hyperactive *hF9*^*R338L*^ variant, effective knock-in was achieved with vector input comparable to that commonly used in clinics. Germline safety, off-target effect, and anti-Cas9 immunity were also evaluated to address the potential concerns in developing somatic knock-in gene therapies.

## Results

### A single dose of AAV-CRISPR rendered stable NHEJ knock-in of ires-*hF9* and long-term hemostasis correction in adult hemophilia B mice

First, we compared in vivo NHEJ knock-in mediated by Cas9 derived from *Streptococcus pyogenes* (SpCas9) and *Staphylococcus aureus* (SaCas9) through hydrodynamic injection. Previously developed single-cut ires-GFP reporter^12^ was used to avoid vector-based expression, and donor insertion was directed at *mAlb* 3′UTR (Supplementary Fig. 1a). The knock-in using either SpCas9 or SaCas9 in combination with high-performing sgRNAs yielded high intensity of GFP signals in mouse livers (Supplementary Fig. 1b, c). SpCas9/sg-1 (hereafter named as sgAlb) produced the highest GFP signals and was selected for further studies (Supplementary Fig. 1c).

**Fig. 1:**
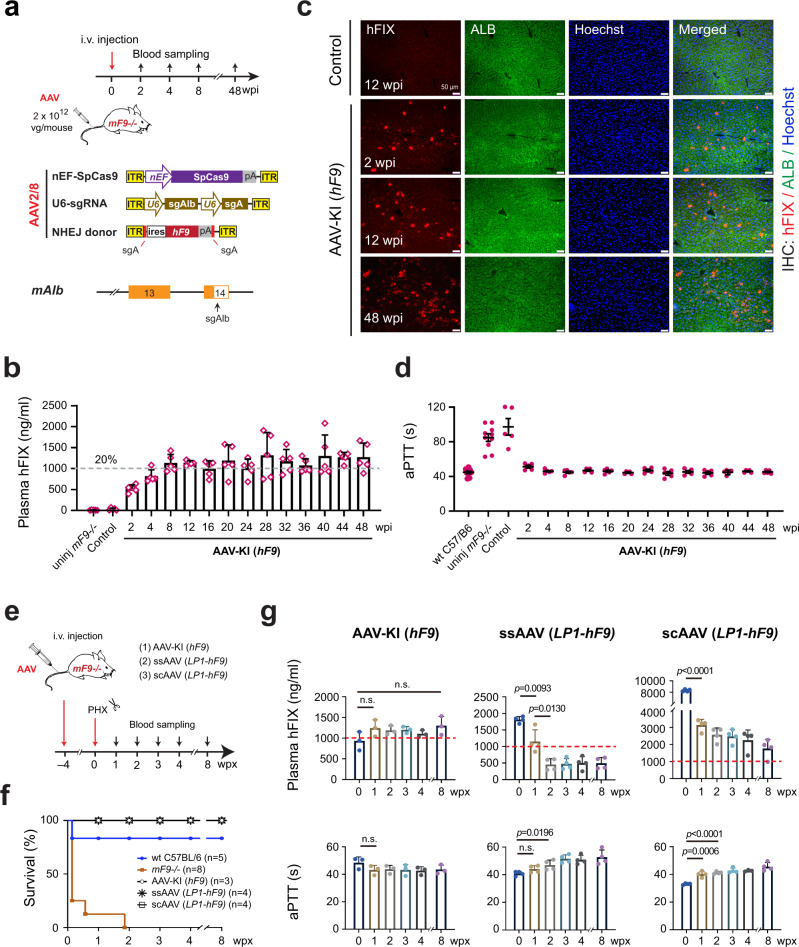
AAV-CRISPR mediated NHEJ knock-in at proximal 3′UTR in *mAlb* locus supported long-term hFIX production and hemostasis correction in adult hemophilia B mice. **a** Schematics for AAV injection into adult *mF9−/−* mice and vector designs. Upper: diagram for the experimental flow. Middle: AAV vectors carrying SpCas9, sgAlb/sgA, and ires-*hF9*. The ires-*hF9* donor was scAAV vector and it carried flanking sgA target sequences. Lower: sgAlb target site at proximal *mAlb* 3′UTR. The total AAV input dose was 2 × 10^12^ vg/mouse, consisting of ires-*hF9* donor (1 × 10^12^), SpCas9 (5 × 10^11^), and sgAlb/sgA (5 × 10^11^) at a ratio of 2:1:1. The treated mice were termed as AAV-KI (*hF9*) mice hereafter. **b** hFIX protein levels in AAV-KI (*hF9*) mouse plasma detected by ELISA. The Control group was injected with ires-*hF9* donor only. The data for AAV-KI (*hF9*) group were collected every 2–4 weeks until 48 wpi (*n* = 5). Dashed lines represent 20% of normal FIX levels. Data are mean ± SD. **c** IHC staining on livers from AAV-KI (*hF9*) mice at 2, 12, and 48 wpi. Hepatocytes were stained with antibodies specific to hFIX (red) and mouse ALB (green). Nuclei were counterstained with Hoechst (blue). Bar = 50 µm. **d** Plasma aPTT values in wt C57BL/6 (*n* = 10), uninjected *mF9−/−* mice (*n* = 10), control group (*n* = 5) and AAV-KI (*hF9*) mice (*n* = 5). Each data point represents an individual mouse. The data for AAV-KI (*hF9*) group were collected every 2–4 weeks until 48 wpi. Data are mean ± SD. **e** Schematic diagram for the experimental flow. *mF9−/−* mice were i.v. injected with either AAV-KI (*hF9*) cocktail, ssAAV (*LP1*-*hF9),* or scAAV (*LP1*-*hF9)*, followed by partial hepatectomy (PHX). wpx: weeks post PHX. **f** Survival rates after PHX surgeries in mice shown in (**e**). wt C57BL/6 and uninjected *mF9−/−* were treated with PHX as controls. The number of mice per group was indicated in the graph legend. **g** Plasma hFIX levels (upper panels) and aPTT values (lower panels) in mice shown in (**e**). Dashed lines represent 20% of the normal plasma FIX level. Data are mean ± SD. Statistical analyses were performed using two-tailed unpaired *t*-tests. n.s. not significant.

Next, we constructed an AAV donor carrying ires-*hF9* to evaluate the therapeutic potential of AAV-CRISPR mediated in vivo NHEJ knock-in strategy. Self-complementary AAV (scAAV) vector was used to provide better intracellular stability^18^, and flanking sgA target sequences were used to introduce DSBs in the donor to facilitate NHEJ knock-in^12^. Recombinant vectors carrying ires-*hF9*, SpCas9, and sgAlb/sgA were encapsulated in AAV8 capsid and intravenously (i.v.) injected into hemophilia B mice (*mF9−/−*)^19^ (Fig. 1a). The treated *mF9−/−* mice (6–12 weeks), named AAV-KI (*hF9*) mice hereafter, produced high levels of circulating hFIX in the plasma, which increased steadily during the first 8-wpi and maintained at 1000–1300 ng/ml until the mice were sacrificed at 48 wpi (Fig. 1b). Immunohistochemistry (IHC) and quantitative reverse transcription polymerase chain reaction (qRT-PCR) analyses of liver tissues collected at 2, 12, and 48 wpi confirmed the persistent *hF9* expression in hepatocytes throughout the whole observation period (Fig. 1c and Supplementary Fig. 2a).

The hemostasis activity of AAV-KI (*hF9*) mice was assessed by measuring activated partial thromboplastin time (aPTT). Indeed, aPTT values in AAV-KI (*hF9*) mice returned to normal and remained stable throughout the 48-week observation period (Fig. 1d), indicating long-term correction of hemostasis. No apparent liver damage was observed (Supplementary Fig. 2b, c).

To assess the genetic stability of *hF9* knock-in in fast-growing tissues, partial hepatectomy (PHX) was performed on AAV-KI (*hF9*) mice, and hFIX production was measured during the recovery period (Fig. 1e). To differentiate the knock-in effect from non-integrative AAV expression, PHX was also performed on mice receiving ssAAV and scAAV bearing LP1 promoter-driven *hF9* (Fig. 1e). Uninjected *mF9−/−* mice suffered from excessive bleeding and had a low survival rate within one-week post-PHX (wpx). In contrast, *mF9−/−* mice that received *hF9* transgene by either knock-in or episome strategies showed 100% survival (Fig. 1f). Interestingly, plasma hFIX levels in AAV-KI (*hF9*) mice increased slightly after PHX. Whereas ssAAV (*LP1*-*hF9)* and scAAV (*LP1*-*hF9)* treated mice showed a sharp reduction in plasma hFIX levels after PHX (Fig. 1g, upper panels), indicating dilutions of episomal AAVs during liver regeneration^20,21^. After PHX, the aPTT values remained low among all *hF9*-treated mice, except a slight increase in ssAAV (*LP1*-*hF9)* and scAAV (*LP1*-*hF9)* groups (Fig. 1g, lower panels), which correlated with the drastic reduction of plasma hFIX levels. At 8 wpx, substantial hFIX production was detected in all hFIX-treated mice (Supplementary Fig. 3), which explained their survival post-PHX surgery.

### AAV-CRISPR mediated *hF9* knock-in resulted in subtle transcriptome alteration and off-target effect in mouse liver

**Fig. 2:**
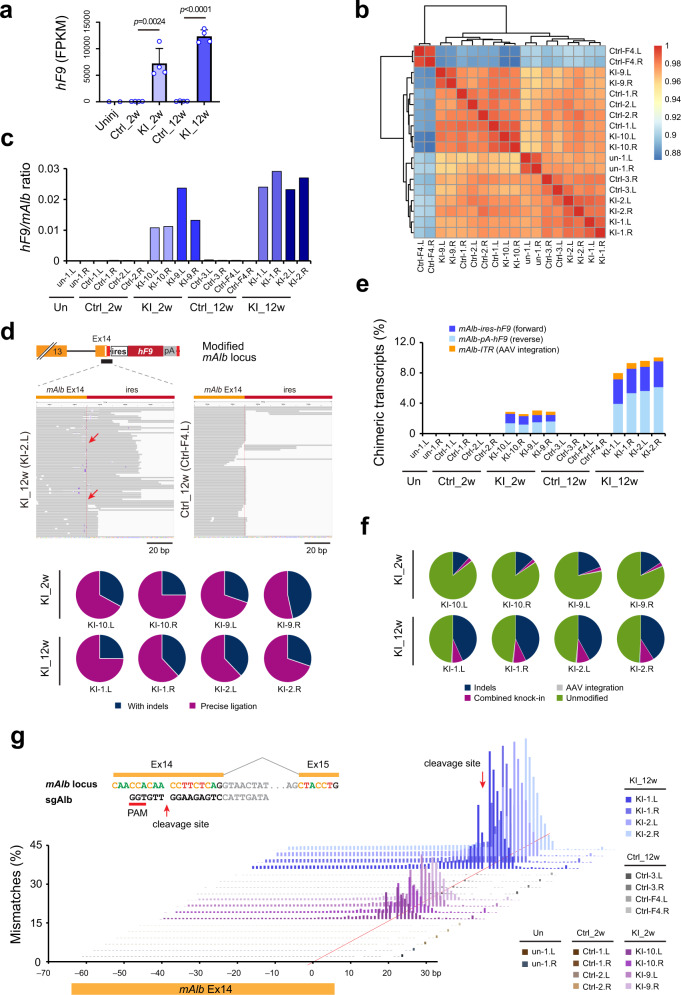
RNA-seq analysis confirmed NHEJ knock-in of *hF9* at *mAlb* 3′UTR and showed no transcriptome alternation. **a** Levels of *hF9* transcripts detected by RNA-seq in livers of uninjected (*n* = 2), control (*n* = 4) and AAV-KI (*hF9*) mice (*n* = 4) at 2 wpi and 12 wpi. Data are mean ± SD. Statistical analyses were performed using two-tailed unpaired *t*-tests. FPKM: fragments per kilobase million. **b** Transcriptomic correlation heat map built with *R* values based on the 18 RNA-seq samples (Supplementary Fig. 4a). Data are Pearson correlation coefficients. RNA samples from the left and right halves of the liver from each mouse were labeled as “L” and “R”. un: uninjected *mF9−/−* mice; Ctrl: control mice injected with AAV carrying ires-hF9 donor only; KI: AAV-KI (*hF9*) mice. **c** Ratios of *hF9*:*mAlb* transcripts (FPKM) in each liver RNA sample. **d** Detection of *mAlb-ires-hF9* chimeric transcripts. Upper panels: the representative data from KI-2.L and Ctrl-F4.L, showing alignments of RNA-seq reads from AAV-KI (*hF9*) mice (left) but not control mice (right) mapped to *mAlb-hF9* chimeric transcript template. Red lines indicated the integration junctions. Red arrows indicated junction sequences matching to precise NHEJ ligation. Lower panels: pie charts showing the percentages of *mAlb-ires-hF9* transcripts with precise NHEJ ligation or containing indels, among AAV-KI (*hF9*) mice at 2 wpi (upper row) and 12 wpi (lower row). **e**, The frequencies of chimeric transcripts carrying forward or reverse ires-*hF9*, or with AAV vector integration at the sgAlb target site. **f** The percentages of transcripts carrying indels, targeted insertions (in either orientation), and AAV integrations at the sgAlb target site. Data shown in **e**, **f** are percentages among the total *mAlb*-derived transcripts, calculated based on the RNA-reads mapped to the target site or integration junctions, using the Integrative Genomics Viewer (IGV). **g** The percentages of mismatches at individual nucleotide positions. The sgAlb target sequence in *mAlb* locus and CRISPR cleavage site was shown in the upper left. Intron sequences in the genome were in gray. sgAlb and PAM sequence was in black. Data (lower panel) shown are the nucleotide mismatches between −65 to +25 bp from the sgAlb cleavage site, covering the entire *mAlb* Ex14.

Liver RNA samples collected from uninjected, control, and AAV-KI (*hF9*) mice sacrificed at 2 wpi and 12 wpi were analyzed by deep sequencing (Supplementary Fig. 4a). High levels of *hF9* transcripts were detected in AAV-KI (*hF9*) livers with 7213 ± 2458 FPKM at 2 wpi and 12,333 ± 1081 FPKM at 12 wpi (Fig. 2a), whereas levels of *hF9* transcripts in control livers were negligible. Transcriptome profiles of 18 samples from AAV-KI (*hF9*), control, and untreated livers were found to be highly similar, with correlation *R* values greater than 0.96 among the 16 samples collected from male mice. Only the two samples from a female control mouse showed relatively distinct features (*R* > 0.88) (Fig. 2b; Supplementary Fig. 4b). These results indicated that transducing SpCas9/sgRNA, gene knock-in at *mAlb* 3′UTR, and ectopic *hF9* expression did not cause major functional alteration in the livers.

Since *hF9* transgene was transcribed together with endogenous *mAlb*, we evaluated the knock-in frequency by calculating the ratio of *hF9*:*mAlb* transcripts, which was 1.47 ± 0.53% at 2 wpi and it increased to 2.58 ± 0.24% at 12 wpi (Fig. 2c). Consistently, RNA-seq reads from AAV-KI (*hF9*) livers can be mapped to the integration junction site in *mAlb-ires-hF9* chimeric transcript template, which was absent in control and uninjected mice (Fig. 2d, upper panels). Markedly, many of these reads carried precise fusion sequences of donor and genome at the CRISPR-cleavage sites (Fig. 2d, lower panels). Based on the junction data, 1.04 ± 0.14% *mAlb-ires-hF9* chimeric transcripts were observed at 2 wpi, and the percentage increased to 3.26 ± 0.09% at 12 wpi (Fig. 2e; Supplementary Fig. 5).

As NHEJ knock-in is nondirectional^12^, reverse insertions of ires-*hF9* were also detected specifically in AAV-KI (*hF9*) livers, at comparable levels to those with forwarding insertions, around 1.41 ± 0.15% at 2 wpi and 5.25 ± 0.81% at 12 wpi (Fig. 2e, Supplementary Fig. 5). Whereas AAV vector integrations at sgAlb target site were detected at much lower rates, around 0.38 ± 0.14% and 0.72 ± 0.12% at 2 wpi and 12 wpi, respectively (Fig. 2e, Supplementary Fig. 5). Restricted by the 150-bp length for paired reads, the AAV vector types and integration orientations could not be accessed. These data indicated that cleaved AAV donors are more suitable for NHEJ knock-in.

Consistently, the occurrence of indels was solely observed around sgAlb cleavage site across the entire *mAlb* transcript in AAV-KI (*hF9*) livers. Approximatively, 14.91 ± 2.85% indels were detected at 2 wpi and the rates increased to 42.3 ± 0.89% at 12 wpi (Fig. 2f). Mismatch analysis at individual nucleotide positions indicated that on-target indels largely occurred at SpCas9/sgAlb cutting site between the third and fourth nucleotide positions from protospacer adjacent motif sequence (Fig. 2g).

Furthermore, we assessed the off-target effect using the RNA-seq data. While extensive in silico prediction identified 129 candidate off-target sequences with ≤4 mismatches to the sgAlb target sequence (Supplementary Figs. 6 and 7a), only three were located in exons or in proximity (±50 bp) to exons (Supplementary Fig. 7b). The corresponding coding genes showed low expressions (<2 FPKM) and no correlation with AAV or knock-in treatments (Supplementary Fig. 7c).

We also examined the top ten off-target sequences predicted with Cas-OFFinder by low-throughput genome PCR-sequencing, followed by Inference of CRISPR Edits (ICE) analysis (Supplementary Fig. 8a). Among four liver samples examined, no editing event was observed at any of the candidate off-target sites. In contrast, 13.5 ± 4.5% and 25.5 ± 3.5% indels were observed at sgAlb cleavage site in AAV-KI (*hF9*) liver DNAs collected at 12 wpi and 48 wpi, respectively (Supplementary Fig. 8b, c).

### NHEJ knock-in of *hF9* in neonatal hemophilia B mice corrected hemostasis without altering the germline genome

**Fig. 3:**
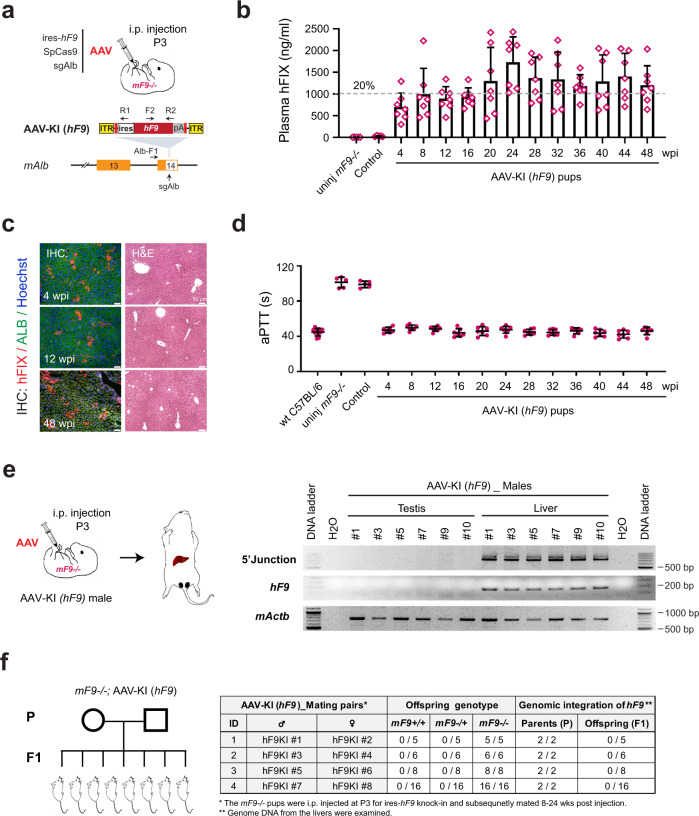
NHEJ knock-in of *hF9* in neonatal mice corrected hemophilia B without modifying germline genome. **a** Schematics for i.p. injection of AAVs carrying SpCas9, sgAlb/sgA and ires-*hF9* into neonatal *mF9−/−* pups at postnatal day 3 (P3) (upper), AAV vector carrying ires-*hF9* (middle) and the sgAlb target site at *mAlb* 3’UTR (lower). **b** hFIX protein levels in pup plasma after i.p. injection of AAVs carrying SpCas9 (1 × 10^11^ vg/mouse), sgAlb/sgA (1 × 10^11^ vg/mouse), and ires-*hF9* donor (2 × 10^11^ vg/mouse) (*n* = 7). The Control group was injected with ires-*hF9* donor only (*n* = 5), and uninjected *mF9−/−* mice were examined as reference (*n* = 5). ELISA was performed using an anti-hFIX antibody, and data for AAV-KI (*hF9*) group were collected every 2–4 weeks until 48 wpi. Dashed lines represent 20% of normal FIX levels. Data are mean ± SD. **c** IHC and H&E staining on livers of AAV-KI (*hF9*) pup sacrificed at 4, 12, and 48 wpi. Hepatocytes were stained using antibodies specific to hFIX (red) and mouse ALB (green). Nuclei were counterstained using Hoechst (blue). Bar = 50 µm. **d** aPTT values of the plasma samples collected from mice shown in (**b**), alongside wt C57BL/6 (*n* = 10). Each data point represents an individual mouse. The data for AAV-KI (*hF9*) pups were collected at different time points until 48 wpi (*n* = 7). Data are mean ± SD. **e** Schematics for injection method and tissue collection after male AAV-KI (*hF9*) pups grew into adults (left). Genome PCR analysis on livers (red) and testes (blue) collected from individual AAV-KI (*hF9*) pups (right). Primer sequences for detecting 5′ junctions (Alb-F1/R1) and *hF9* integration (F2/R2) were listed in Supplementary Table 2. The binding sites of primers Alb-F1/R1 and F2/R2 where indicated in the lower panel in (**a**). **f** Breeding scheme of AAV-KI (*hF9*) pups (left) and genotyping results of the AAV-KI (*hF9*) breeding pairs and their offspring (right).

To investigate the efficacy of NHEJ knock-in in neonatal mice, AAVs at a total dose of 4 × 10^11^ vg/neonate were delivered intraperitoneally (i.p.) into *mF9−/−* pups at P3, and blood samples were collected every 4 weeks (Fig. 3a). The AAV-KI (*hF9*) pups robustly produced hFIX in plasma, which reached 1000–1500 ng/ml by 20 wpi and remained stable until 48 wpi (Fig. 3b). The presence of hFIX was also confirmed by IHC analyses and no sign of liver damage was observed with H&E staining (Fig. 3c). The hFIX-positive hepatocytes in AAV-KI (*hF9)* pups appeared in larger colonies than those in adult mice, implying a greater proliferation rate in young liver cells. Consistently, hemostasis correction was confirmed among the AAV-KI (*hF9*) pups and remained stable throughout the observation period (Fig. 3d).

In contrast, scAAV (*LP1*-*hF9*) injection triggered high plasma hFIX production at 1 wpi, followed by a dramatic decline to 4 wpi, suggesting rapid dilution of episomal scAAVs in fast-growing livers (Supplementary Fig. 9a, b). The decline in plasma hFIX corresponded to slightly higher aPTT values observed from 2 to 4 wpi (Supplementary Fig. 9c). The observations were further supported by IHC staining (Supplementary Fig. 9d, e).

AAV2/8 displays a remarkably broad tissue tropism, yet its ability to infect testicular germ cells remains controversial^22,23^. To address this issue, testicular DNA samples from six male AAV-KI (*hF9*) pups were examined by genome PCR. While AAV-CRISPR mediated *hF9* knock-in was robustly confirmed in all six livers, no *hF9* knock-in or AAV integration was observed in testes (Fig. 3e). Furthermore, four pairs of mice grown from AAV-KI (*hF9*) pups were bred and their offspring were examined for possible generational transmission (Supplementary Fig. 10a). Genome PCR analysis showed that F1-generation pups carried neither *hF9* knock-in nor AAV integration (Fig. 3f and Supplementary Fig. 10b). Consistently, their aPTT values were the same as uninjected *mF9−/−* mice (Supplementary Fig. 10c). These data indicated that AAV-CRISPR mediated NHEJ knock-in was restricted to somatic tissues, which posed a minimal risk for germline modification and unintended vertical transmission.

### Liver-specific knock-in via AAV-CRISPR eliminated unintended genome editing in off-target tissues

**Fig. 4:**
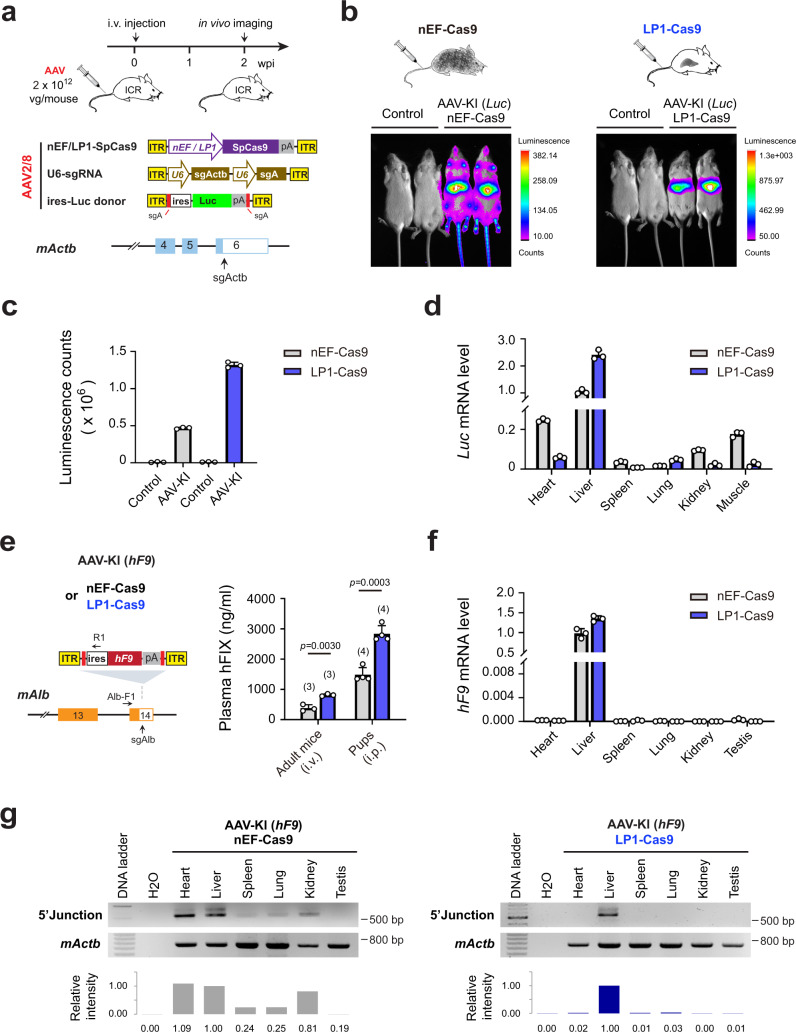
Tissue-specific knock-in mediated by LP1-SpCas9. **a** Schematics for luciferase reporter knock-in at *mActb* locus using nEF- or LP1-SpCas9. Upper: diagram for the experimental flow. Middle: AAV vectors carrying nEF- or LP1-SpCas9, sgActb/sgA, and ires*-*Luc. Lower: sgRNA target sites at *mActb* 3′UTR. **b** Luminescence images of treated ICR mice at 2 wpi. Targeted insertion was mediated by AAV2/8-delivered nEF-SpCas9 (left) or LP1-SpCas9 (right), shown in (**a**). Control mice were injected with AAV carrying ires-luc donor only. **c** Luminescence counts in livers of mice shown in (**b**). Data are mean ± SD (*n* = 3). **d** The *Luc* mRNA levels among different tissues in ICR mice are shown in (**b**). The mice were sacrificed at 2 wpi after i.v. injection of AAVs carrying ires-*Luc* donor (1 × 10^12^ vg/mouse), sgActb/sgA (5 × 10^11^ vg/mouse), together with either nEF-SpCas9 (gray bars) or LP1-SpCas9 (dark blue bars) (5 × 10^11^ vg/mouse). qRT-PCR data shown are as average ± SD (*n* = 3). **e** Schematics of *hF9* knock-in at *mAlb* locus using nEF- or LP1-SpCas9 (Left) and plasma hFIX levels of treated mice sacrificed at 2 wpi (Right). Adult *mF9−/−* mice at 8–12 weeks (adults, *n* = 3) were i.v. injected with AAVs at total 2 × 10^12^ vg/mouse; while the neonates at P1 (pups, *n* = 4) were i.p. injected with AAVs at total 4 × 10^11^ vg/pup. Data are mean ± SD. **f** The *hF9* mRNA levels among different tissues of mice in (**e**), qRT-PCR data are shown as average ± SD (*n* = 3). **g** Genome PCR detection of *hF9* knock-in among different tissues of mice in (**e**), at 2 wpi after i.v. injection with either nEF-Cas9 (left) or LP1-Cas9 (right). The binding sites of primers Alb-F1/R1 were indicated in the left panel in (**e**). Relative intensities of the PCR-amplified fragments were measured using Image J.

In preceding experiments, liver-specific expression was established in mice by knocking-in promoterless transgenes at *mAlb* locus (Figs. 1–3), where AAV2/8-delivered nEF-SpCas9 could silently edit the host genome in other tissues. We then replaced the universal nEF promoter with a liver-specific LP1 promoter to restrict knock-in activity to the liver. To visualize the tissue-specific knock-in, AAV donor carrying luciferase reporter was knocked-in at the ubiquitously expressed *mActb* at 3′UTR using either LP1-SpCas9 or nEF-SpCas9 (Fig. 4a). Gene knock-in using nEF-SpCas9 produced broad luciferase signals throughout the body (Fig. 4b, left). In contrast, LP1-SpCas9 mediated tissue-specific editing, yielding signals exclusively restricted to the liver (Fig. 4b, right). Noticeably, the high promoter activity of LP1 also contributed to higher luciferase activity in AAV-KI (Luc) mouse livers (Fig. 4c,d).

Next, we compared LP1-SpCas9 and nEF-SpCas9 mediated *hF9* knock-in at *mAlb* 3′UTR in *mF9−/−* mice. Similarly, higher hFIX production was detected in both adult and neonatal AAV-KI (*hF9*) mice treated with LP1-SpCas9 (Fig. 4e). Although site-specific knock-in at *mAlb* resulted in liver-specific *hF9* expression in both LP1-SpCas9 and nEF-SpCas9 edited mice (Fig. 4f), genome PCR analysis confirmed that targeted insertion of *hF9* was confined to the liver in mice edited using LP1-SpCas9, whereas those treated with nEF-SpCas9 received *hF9* knock-in in multiple organs including heart and kidney (Fig. 4g).

### Liver-specific NHEJ knock-in corrected hemostasis in neonates and adults with low-dose AAV

**Fig. 5:**
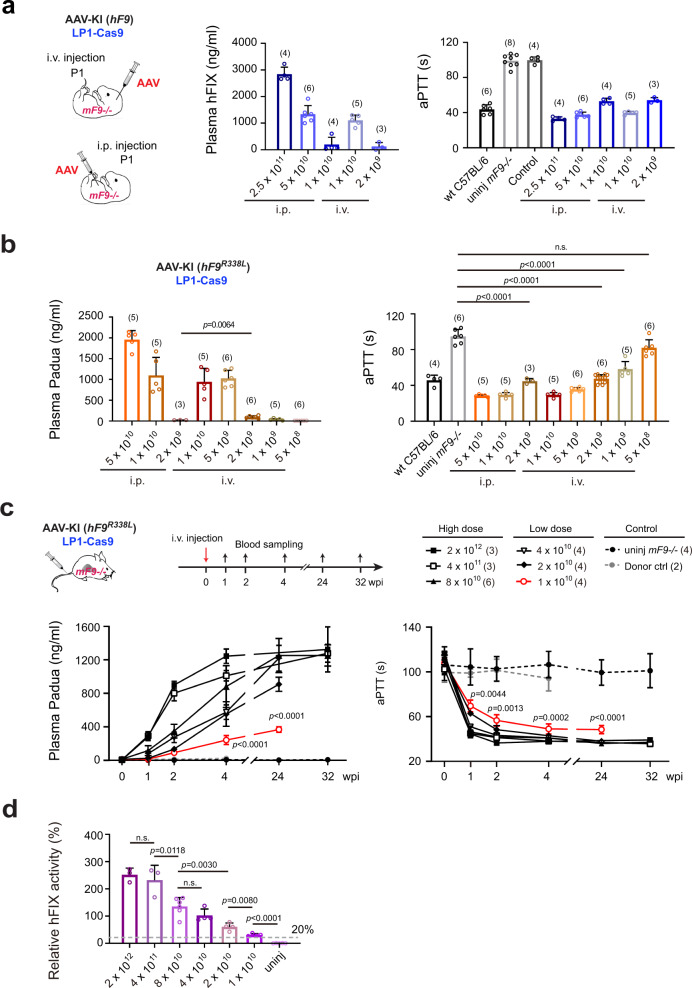
Liver-specific knock-in of *hF9*^*R338L*^ with low-dose AAV inputs corrected hemostasis in *mF9−/−* neonates and adults. **a** Schematic diagram showing i.v. or i.p. injection of AAVs in *mF9−/−* neonates for LP1-Cas9 mediated *hF9* knock-in (left). *mF9−/−* neonates were injected at P1 with AAV2/8 carrying ires-*hF9* donor, LP1-SpCas9 and sgAlb/sgA at 2:1:1 ratio. Plasma hFIX levels (middle) and aPTT values (right) were analyzed at 2 wpi. Total AAV doses (vg/mouse) used in each group were indicated in the *x*-axis. The number of mice per group (*n*) was indicated in the brackets above the data bars. **b** Knock-in of *hF9*^*R338L*^ in *mF9−/−* neonates with various AAV doses. *mF9−/−* neonates were injected at P1 with AAV2/8 carrying ires-*hF9*^*R338L*^ donor, LP1-SpCas9 and sgAlb/sgA at 2:1:1 ratio. Plasma hFIX-Padua levels (left) and aPTT values (right) were measured at 2 wpi. Total AAV doses (vg/mouse) used in each group were indicated in the *x*-axis. The number of mice per group (*n*) was indicated in the brackets above the data bars. **c** Knock-in of *hF9*^*R338L*^ in adult *mF9−/−* mice with various AAV doses. *mF9−/−* mice at 6–12 weeks were injected with AAV2/8 carrying ires-*hF9*^*R338L*^ donor, LP1-SpCas9, and sgAlb/sgA at 2:1:1 ratio. Control mice were injected with donors only. The schematics for i.v. injection and workflow were shown in the upper left. The AAV doses (vg/mouse) tested were shown in the graph legend (upper right). The number of mice per group (*n*) was indicated in the brackets after the doses. Data shown were plasma hFIX-Padua levels (lower left) and aPTT values (lower right) measured at 0, 1, 2, 4, 24, and 32 wpi for individual mouse groups. **d** Relative hFIX activities at 2 wpi among different dose groups in (**c**). The AAV doses (vg/mouse) used in each group were indicated in the *x*-axis. The standard curve was generated using recombinant human factor IX (MonoFIX). The number of mice per group (*n*) was indicated in the brackets after the doses in (**c**). All data are mean ± SD. Statistical analyses were performed using two-tailed unpaired *t*-tests. n.s. not significant.

To determine the lowest AAV dose needed for effective *hF9* knock-in in neonatal mice, the therapeutic AAV mixture consisted of ires-*hF9* donor, LP1-SpCas9, and sgAlb/sgA at 2:1:1 ratio was successively diluted in 1:5 with PBS and delivered into *mF9−/−* neonates at P1 through i.p. or i.v. routes (Fig. 5a, left). Blood assays at 2 wpi showed that the lowest dose which yielded significant hFIX production and corrected hemostasis was 1 × 10^10^ vg/neonate via i.p injection and 2 × 10^9^ vg/neonate via i.v. injection. More than 100 ng/ml of plasma hFIX was detected and a significant reduction in aPTT value was observed in these mice (Fig. 5a, middle and right).

Next, we constructed an AAV donor carrying *hF9*^*R338L*^ mutant, which encodes hyperactive hFIX variant Padua^24^, to explore a further reduction of AAV dose needed for therapeutic knock-in. The ires-*hF9*^*R338L*^ donor was delivered alongside LP1-SpCas9 and sgAlb/sgA at a 2:1:1 ratio into *mF9−/−* neonates at P1 through i.p. or i.v. routes. Plasma assays at 2 wpi confirmed superior coagulation activity of hFIX-Padua (Fig. 5b). Hemostasis correction through i.p. injection was achieved with AAV dose as low as 2 × 10^9^ vg/neonate (Fig. 5b, right), approximatively fivefold lower than those using normal *hF9* (Fig. 5a). Notably, with the same AAV dose at 2 × 10^9^ vg/neonate, i.v. administration resulted in a higher plasma Padua level and significantly lower aPTT value than those of i.p. injection (Fig. 5b), yielding robust hFIX activity and persistent hemostasis correction till 20 wpi (Supplementary Fig. 11).

To further evaluate the dose effect, we conducted a dose-response assay for *hF9*^*R338L*^ knock-in in adult *mF9−/−* mice. The AAV mixture containing ires-*hF9*^*R338L*^, LP1-SpCas9, and sgAlb/sgA was successively diluted to yield six different doses: 2 × 10^12^, 4 × 10^11^, 8 × 10^10^, 4 × 10^10^, 2 × 10^10^, 1 × 10^10^ vg/mouse (Fig. 5c, upper). 6–12-week *mF9−/−* mice were divided into six groups to receive the different doses of AAVs through i.v. injection. Blood samples were collected weekly or biweekly up to 32 wpi. Notably, mice in all six treatment groups produced plasma hFIX-Padua at significant levels. Similar to the observations in adult AAV-KI (*hF9*) mice (Fig. 1b), a steady increase of plasma hFIX-Padua in the first 4–6 wpi was evident in all six groups (Fig. 5c, lower left). Effective hemostasis correction was achieved with AAV dose as low as 1 × 10^10^ vg/mouse, which produced plasma hFIX-Padua at around 350 ng/ml (Fig. 5c, lower, red circle). The relative hFIX activities among all treatment groups were restored significantly since 2 wpi, with the lowest AAV dose at 1 × 10^10^ vg/mouse yielding approximately 30% of the normal hFIX activity (Fig. 5d).

### In vivo knock-in with low-dose AAV-CRISPR greatly reduced indels at the target site among neonatal mice

**Fig. 6:**
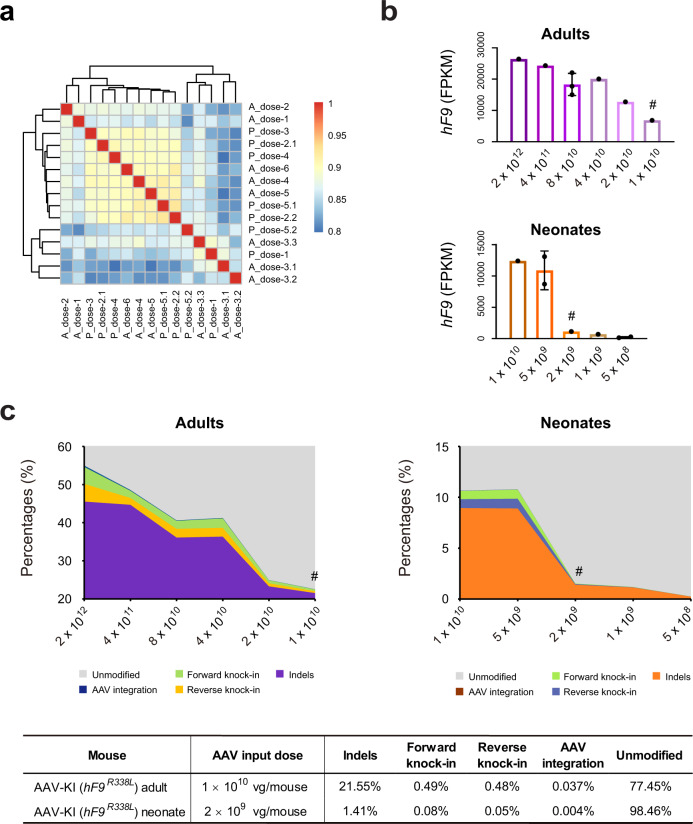
RNA-seq analysis for liver-specific knock-in of *hF9*^*R338L*^ with various AAV input doses revealed much-reduced indel rates at a target site in neonatal mice. **a** Transcriptomic correlation heat map built with *R* values based on RNA-seq data of 15 liver samples collected from adult and neonatal AAV-KI (*hF9*^*R338L*^) mice receiving various AAV doses. Data are Pearson correlation coefficients. Samples and treatment conditions were shown in Supplementary Fig. 12. **b** Levels of *hF9*^*R338L*^ transcripts detected by RNA-seq among the adult (upper) and neonatal (lower) AAV-KI (*hF9*^*R338L*^) liver samples examined in (**a**). Each dot represents one mouse sample. Data shown are mean ± SD for adult mice receiving 8 × 10^10^ vg/mouse (*n* = 3) and neonates receiving 5 × 10^9^ and 5 × 10^8^ vg/pup (*n* = 2). Data at other doses were from one mouse (*n* = 1). FPKM fragments per kilobase million. #: the minimum effective AAV input doses yielding relative hFIX activity above 20% of the normal (Fig. 5d; Supplementary Fig. 11b). **c** Percentages of *mAlb* transcripts carrying indels, forward or reverse ires-*hF9*^*R338L*^, and AAV integration at sgAlb target site, among the adult (upper left) and neonatal (upper right) AAV-KI (*hF9*^*R338L*^) mice examined in (**a**). The AAV doses (vg/mouse) used in each group were indicated in the *x*-axis. #: mice treated with minimum effective AAV input doses. The representative data in the table (lower) are from adult and neonatal AAV-KI (*hF9*^*R338L*^) mice treated with minimum effective AAV input doses (marked with # in upper panels).

To elucidate the in vivo gene editing profiles yielded with low-dose AAV-CRISPR, liver RNAs extracted from fifteen AAV-KI (*hF9*^*R338L*^) mice receiving various AAV doses at either adult or neonatal stage were analyzed by deep sequencing (Supplementary Fig. 12). Regardless of treatment time points and AAV input doses, the transcriptome profiles were highly similar (*R* ≥ 0.8) among all the samples examined (Fig. 6a). The levels of *hF9*^*R338L*^ transcripts in both adult and neonatal AAV-KI (*hF9*^*R338L*^) were AAV dose-dependent (Fig. 6b), which were concordant with the plasma hFIX-Padua concentrations (Fig. 5b, c).

Subsequently, the frequencies of indels, forward and reverse insertions of ires-*hF9*^*R338L*^, and AAV integrations at sgAlb target site were assessed. Based on RNA reads mapped to knock-in/integration junctions, ires-*hF9*^*R338L*^ insertion in either orientation decreased with reduced AAV input (Fig. 6c). In the adult mouse receiving AAV input at 1 × 10^10^ vg/mouse, the forward and reverse knock-in was 0.49% and 0.48%, respectively. Whereas in the neonatal mouse treated with the lowest effective AAV dose at 2 × 10^9^ vg/neonate, 0.077% forward and 0.052% reverse insertions were detected. The AAV integrations were around 0.037% and 0.004% in the corresponding adult and neonatal AAV-KI (*hF9*^*R338L*^) mouse examined (Fig. 6c).

Interestingly, rates of indels at sgAlb target site were significantly lower in neonatal AAV-KI (*hF9*^*R338L*^) mice, compared to their adult counterparts while AAV dose-dependent reduction was observed in both groups (Fig. 6c). The adult AAV-KI (*hF9*^*R338L*^) mouse receiving AAV input at 1 × 10^10^ vg/mouse yielded approximate 21.55% of *mAlb* transcripts carrying on-target indels. Distinctly, 6.43%–11.39% indels were observed in the neonatal mice treated with high AAV doses at 1 × 10^10^ and 5 × 10^9^ vg/neonate, while only around 1.41% indels were detected when AAV input was reduced to 2 × 10^9^ vg/neonate.

### AAV-CRISPR induced heterogeneous anti-Cas9 immunities in adult mice, but low immunity to Cas9 in neonatal mice

**Fig. 7:**
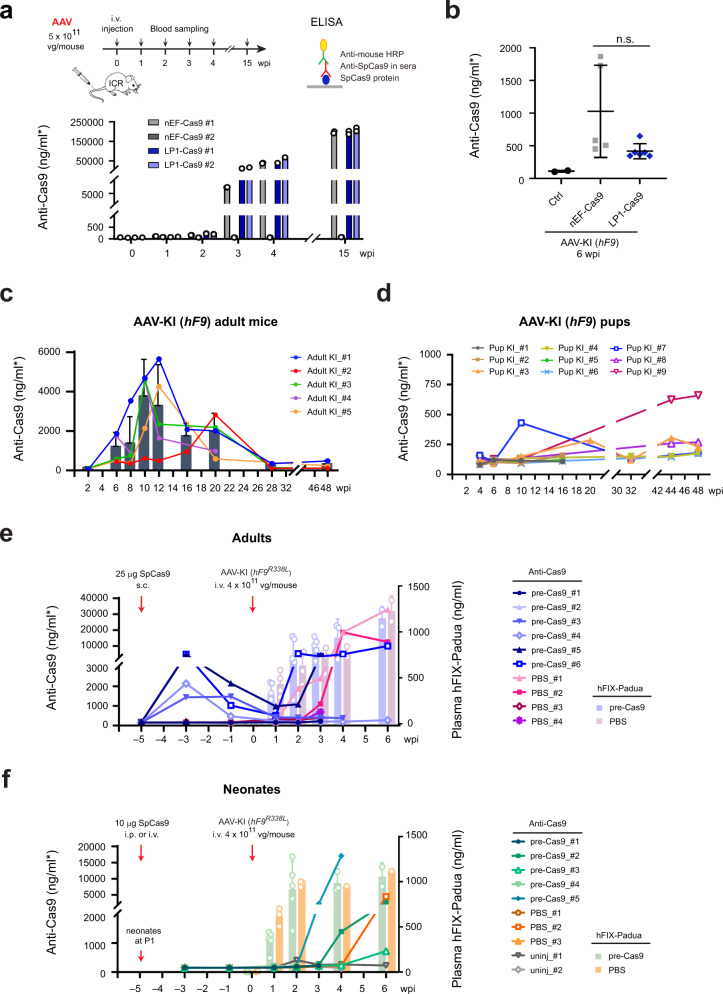
AAV-CRISPR induced heterogeneous anti-Cas9 immunities in adult mice, but low anti-Cas9 immunity in neonatal mice. **a** Workflow for i.v. injection of AAVs carrying nEF-Cas9 or LP1-Cas9 into adult ICR mice (upper left) and schematics for ELISA assay using mouse plasma for anti-Cas9 antibody detection (upper right). The data showed plasma levels of anti-Cas9 antibodies detected at 1, 2, 3, 4, and 15 wpi (Lower). Each bar indicates one mouse. Data are mean ± SD for replicate measurements (*n* = 2). **b** Plasma levels of anti-Cas9 antibodies in AAV-KI (*hF9*) mice edited using nEF-Cas9 (*n* = 5) or LP1-Cas9 (*n* = 5) in Fig. 4e. Each dot represents one mouse sacrificed at 6 wpi. Ctrl: control mice injected with ires-*hF9* donor only (*n* = 2). Data are mean ± SD. Statistical analyses were performed using two-tailed unpaired *t*-tests. n.s. not significant. **c** Retro-analysis showing levels of plasma anti-Cas9 antibodies in adult AAV-KI (*hF9*) mice in Fig. 1b–d. Each line represents one mouse. Data bars at each time point represent mean ± SD (*n* = 5). **d** Retro-analysis showing the levels of plasma anti-Cas9 antibodies in AAV-KI (*hF9*) pups in Fig. 3b–d. Each line represents one mouse. **e** Levels of anti-Cas9 antibody (left *y*-axis) and hFIX-Padua (right *y*-axis) in SpCas9-immunized adult *mF9−/−* mice before and after receiving AAV-KI (*hF9*^*R338L*^). Data for anti-Cas9 antibodies were indicated using lines, and each line represents one mouse. Plasma hFIX-Padua concentrations from pre-immunized mice (*n* = 6) and the control group (*n* = 4) were shown at each time point, in light blue and light pink bars, respectively. Data are mean ± SD. **f** Levels of anti-Cas9 antibody (left *y*-axis) and hFIX-Padua (right *y*-axis) in SpCas9-immunized neonatal *mF9−/−* mice before and after receiving AAV-KI (*hF9*^*R338L*^). Data for anti-Cas9 antibodies were indicated using lines and each line represents one mouse. Padua concentrations from pre-immunized mice (*n* = 5) and the control group (*n* = 3) were shown at each time point, in light green and light orange bars, respectively. Data are mean ± SD. Red arrows in **e**, **f** indicate the time points for i.p. or i.v. injection of SpCas9 protein (left) and i.v. injection of AAV mixture (right). AAV input doses used were indicated 638.212.

To address the immunogenicity of AAV-delivered Cas9, we determined the presence of anti-Cas9 antibodies after AAV-CRISPR treatment in various mouse models. Adult ICR mice injected with AAV2/8 carrying either nEF-Cas9 or LP1-Cas9 were sampled weekly or bi-weekly to determine the levels of plasma anti-Cas9 antibody using homemade ELISA^25^ (Fig. 7a, upper). Three out of four mice developed antibodies against Cas9, and the levels increased dramatically after 3 wpi. The remaining one showed negligible anti-Cas9 activity throughout the 15-week observation period (Fig. 7a, lower). Plasma analysis for adult AAV-KI (*hF9*) mice edited with nEF-Cas9 or LP1-Cas9 (Fig. 4e) also showed diverse levels of anti-Cas9 activities at 6 wpi, without significant difference between the two groups (Fig. 7b).

Retro-analysis of adult AAV-KI (*hF9*) mice (Fig. 1b–d) showed detectable plasma Cas9 antibodies in all five mice during 6–20 wpi. While four mice showed the highest anti-Cas9 activity around 10–12 wpi, one mouse responded slowly and its anti-Cas9 activity peaked at 20 wpi. The overall levels of anti-Cas9 activities declined in all five mice after 20 wpi and remained at low levels after 28 wpi (Fig. 7c).

Consistently, heterogenous anti-Cas9 activities were observed in adult AAV-KI (*hF9*^*R338L*^) mice receiving different AAV doses (Supplementary Fig. 13a). Levels of anti-Cas9 antibodies detected at 6 wpi showed no correlation with AAV inputs, hFIX-Padua production, or Cas9 expression levels (Supplementary Fig. 13b, c). In contrast, anti-AAV2/8 antibodies were robustly developed in all treated mice (Supplementary Fig. 14). Collectively, these findings indicated that SpCas9 provoked heterogeneous immune responses in adult mice, which did not cause dramatic elimination of gene-edited hepatocytes.

Interestingly, retro-analysis of AAV-KI (*hF9*) pups (Fig. 3b–d) demonstrated slower development of immune responses and lower anti-Cas9 activities than those in adult mice (Fig. 7d). Similarly, AAV-KI (*hF9*) pups receiving lower AAV doses (Fig. 5a) showed sporadic anti-Cas9 activities, regardless of the administration routes (i.p. or i.v.) (Supplementary Fig. 15a). Markedly, AAV-KI (*hF9*) pups also developed anti-AAV2/8 antibodies at much lower levels than their adult counterparts. The anti-AAV activities were negligible in pups receiving AAVs at 1 × 10^10^ vg/neonate via i.p. or 2 × 10^9^ vg/neonate via i.v. (Supplementary Fig. 15b). These results suggested that administration at the neonatal stage might bypass the pre-existing immunity and is promising for AAV-CRISPR-based gene-editing therapy.

### Pre-existing anti-Cas9 immunity had a subtle impact on in vivo knock-in by AAV-CRISPR

To evaluate the impact of host anti-Cas9 immunity on AAV-CRISPR mediated in vivo knock-in, we performed *hF9*^*R338L*^ knock-in in pre-immunized mice. Adult *mF9−/−* mice at 6–12 weeks were pre-immunized with SpCas9 protein at 25 μg/mouse via subcutaneous (s.c.) injection. Neonatal *mF9−/−* pups at P1 were treated with SpCas9 protein at 10 µg/pup by either i.v. or i.p. injection. Heterogeneous anti-Cas9 activities were detected in SpCas9-immunized adult mice, which reached the highest levels after two weeks and decreased substantially at the fifth-week post-immunization (Fig. 7e). In pre-immunized neonatal mice, low anti-Cas9 activities were detected throughout the 5-week observation, regardless of injection routes (Fig. 7f; Supplementary Fig. 16a).

The Cas9-immunized mice and controls were then i.v. injected with AAV mixture containing ires-*hF9*^*R338L*^ donor, LP1-SpCas9, and sgAlb/sgA at 2:1:1 ratio, at a total dose of 4 × 10^11^ vg/mouse. Notably, all mice treated for AAV-KI (*hF9*^*R338L*^) produced plasma hFIX-Padua at significant levels with a steady increase at early stages (Fig. 7e, f). No significant difference was observed between the controls and SpCas9-immunized mice, and similar levels of plasma hFIX-Padua were detected in adult and neonatal pre-immunized mice (Fig. 7e, f). The anti-Cas9 analysis detected no immunological memory in both adult and neonatal mice after SpCas9 immunization (Fig. 7e, f). No reverse correlation was observed between the levels of anti-Cas9 antibodies and plasma hFIX-Padua concentrations, either before or after *hF9*^*R338L*^ knock-in (Supplementary Fig. 16b, c). Collectively, these data suggested that the host anti-Cas9 immunity posed a subtle impact on in vivo gene knock-in outcomes through systemic delivery.

## Discussion

In this study, we demonstrated the use of AAV2/8-delivered CRISPR/SpCas9 for NHEJ-mediated knock-in of *hF9* gene into somatic tissues of the adult and neonatal mice. The efficient targeting at the proximal *mAlb* 3′UTR and high *hF9* expression rendered robust and persistent hFIX production and corrected hemostasis in both adult and neonatal *mF9−/−* mice during the entire 48-week study period. The efficacy of *hF9* knock-in was also confirmed in rapidly growing livers of neonates and partially hepatectomized adult mice. No germline modification or off-target editing was detected. Serum assays and transcriptome analysis showed no evident alteration of liver functions. Furthermore, using LP1-SpCas9 could confine the transgene knock-in to the liver, achieving tissue-specific gene editing. Notably, employing LP1-SpCas9 to knock-in hyperactive *hF9*^*R338L*^ could reduce the AAV input dose to 2 × 10^9^ vg/neonate and 1 × 10^10^ vg/adult for hemostasis correction in *mF9−/−* mice. These AAV inputs were 10- to 100-fold lower than those reported^7–10,13–15^, and the treatment in neonates yielded low levels of anti-AAV and anti-Cas9 antibodies. Collectively, our results suggested that therapeutic knock-in can be achieved with low and safe AAV inputs in clinics, and administration at the neonatal stage is a promising strategy to bypass pre-existing immunity to Cas9 and AAV.

To date, the efficacy of somatic gene knock-in has been well demonstrated in preclinical studies^26–28^, and its potential in developing an almost permanent cure was ascertained^16,17^. However, the technologies are still in their infancy to provide new drug candidates, warranting further research to explore various expression strategies and target loci to address the hidden potentials and limitations. Studies have established that inserting a promoterless transgene sequence at a pre-selected genomic locus not only enables cell-type specific expression but also eliminates unintended gene activation caused by random integration^29^. Bicistronic expression with viral 2A-peptide^14,15^ and ectopic splicing linking transgene sequence to endogenous exons^7,27,30^ was often used to render transgene expression upon correct insertion, but they require in-frame insertion and intronic targeting, respectively. Ires-based expression is more suitable for NHEJ knock-in at 3′UTR^12^, yet its potential for in vivo knock-in has not been well examined. While promoterless *hF9* sequences were commonly inserted at *F9* locus to develop gene-editing therapy for hemophilia B^7,9,10^, gene knock-in at *Alb* locus has been actively explored to establish a general platform. The intron-1^27,30^, intron-11 to 13^14,15,26,31^, and exon-14 (Ex14)^15,26^ in *Alb* locus have been investigated for *hF9*, truncated *F8* or *UTG1A1* gene knock-in, yielding desired therapeutic outcomes in both mouse and rat. In this study, we explored *mAlb* 3′UTR covering partial Ex14 and entire Ex15 for *hF9* knock-in, to avoid potential disruption of *mAlb* expression in the edited hepatocytes. Despite several sgRNAs targeting *mAlb* Ex14 being reported^15,26^, we identified three high-performing sgRNAs that were not examined previously (Supplementary Fig. 1). With the best-performing sgAlb cleaving the Ex14 at proximal *mAlb* 3′UTR, we employed ires to express the full-length *hF9* coding sequence for NHEJ knock-in. Moreover, we demonstrated that adopting tissue-specific Cas9 expression could confine the transgene insertion to a designated organ and prevent undesired genome-editing in other tissues (Fig. 4). This platform not only provides better versatility and flexibility but also demonstrates higher liver-specific expression compared to other strategies reported^7,10,27,30^.

The large sizes of Cas9 proteins and a small payload of AAV often require more than one AAV vector for in vivo gene knock-in. While SaCas9 (~3.3 kb) could be delivered alongside with sgRNA cassette^3,9,14^, SpCas9 (~4.2 kb) and its sgRNA need to be packaged into separate AAV vectors^2,15^. In this study, we used two ssAAV vectors to deliver SpCas9 and sgAlb/sgA and one scAAV vector to carry the donor to achieve desired *hF9* knock-in. Notably, the AAV doses used in this approach were significantly lower than those reported. Future research is still warranted to explore the optimal vector ratio, and side-by-side comparison with strategies delivering sgRNA together with *hF9* sequences^27,31^ would be necessary.

The AAV doses formerly reported for effective somatic knock-in were 0.5–5 × 10^12^ vg/adult mouse^7–10,13–15,26,28,30,32^, which correspond to 2.5–25 × 10^14^ vg/kg for humans. The high AAV input dose is not only prohibitively expensive but also highly toxic to the recipients. Severe systemic and neuronal toxicity were observed in non-human primates and pig models using AAVs at the dose of 2 × 10^14^ vg/kg^33^, while fatal sepsis was reported in recent clinical trials involving i.v. administration of AAVs at 3 × 10^14^ vg/kg^34^. Therefore, lowering the AAV input is pivotal to achieving safe somatic knock-in for subsequent clinical translation.

In this study, liver-specific NHEJ knock-in of *hF9*^*R338L*^ at proximal *mAlb* 3′UTR further reduced the AAV inputs required for hemostasis correction to 2 × 10^9^ vg/neonate and 1 × 10^10^ vg/adult mouse. This AAV input is approximately equivalent to broadly adopted doses in clinical trials and is regarded to be safe in humans. Our results demonstrated that a careful selection of Cas9 orthologues, sgRNAs, as well as delivery and expression strategies, could achieve therapeutic efficacy without jeopardizing the patient’s safety with dose-related toxicity. Meanwhile, the longitudinal and dose-response data for *hF9* and *hF9*^*R338L*^ knock-in improved our understanding of controlling AAV-CRISPR mediated gene editing at a minimum effective level. This is crucial to avoid potential side effects, such as venous thromboembolism (VTE), that may be caused by overdose during hemophilia B treatment^35^.

Advancement of gene-editing therapy is hindered by inadvertent genomic modifications. In this study, our data delineated the various editing outcomes and addressed their impacts. First, CRISPR can tolerate minor mismatches, leading to off-target cleavage^36,37^. Yet, targeting specificity could be improved by optimizing the targeting strategies and CRISPR (or ZFN) components^38,39^. In this study, neither genomic nor transcriptional alterations caused by off-target editing were detected (Supplementary Figs. 6–8), supporting that off-target risks can be minimized when high-quality sgRNA and target site were used.

Second, the possibility of germline modification poses ethical concerns, even though the vertical transmission has not been observed during somatic genome editing^23,40^. We verified that the germline genomes of AAV-KI (*hF9*) pups, which were edited with the broadly expressed nEF1-SpCas9 at the neonatal stage, were free from modifications (Fig. 3e, f; Supplementary Fig. 10). Moreover, the use of LP1 promoter confined the gene editing activity to the liver (Fig. 4) and protected the germline genome from unintended editing.

Third, AAV vectors integrate into the somatic genome through DNA capture at double-strand breaks (DSB)^29^, at rates of around 0.05% in neonatal mice and between 10E − 4 and 10E − 5 in non-human primates and humans^41^. Despite tumorigenic potentials associated with random AAV integrations still under debate^42,43^, AAV integration with strong preference at CRISPR (or ZFN) target sites raised another concern for developing AAV-based gene editing therapy^40,44,45^. In this study, RNA-reads carrying *mAlb* Ex14 fused with AAV-ITR were quantified to evaluate AAV vector integrations at the target site (Fig. 2e, f; Supplementary Fig. 5). No other ITR-containing chimeric transcripts were observed, suggesting random AAV integrations were negligible.

Fourth, the NHEJ mechanism mediates knock-in in either orientation^12^. We detected chimeric *mAlb* transcripts carrying reverse ires-*hF9* sequences, which had low coding potential and were subjected to removal by splicing^46^. Interestingly, insertions of sgA-cleaved *ires-hF9*, in either orientation, were detected at much higher rates compared to AAV integrations (Fig. 2e, f; Supplementary Figs. 5 and 6), suggesting that AAV captures at sgAlb target site occurred at a much lower frequency compared to the knock-in of donors with DSBs. Nevertheless, no evident adverse outcomes were associated with reverse knock-in and AAV integration. The underlying molecular mechanisms are largely unexplored, and further reduction of the unintended knock-in/integration through new vector designs would be desired.

Finally, default NHEJ repair generates background indels at the CRISPR cleavage site. Our data showed that the indel frequencies increased over time (Fig. 2e, f) and correlated with AAV inputs (Fig. 6c), ranging from 12.06% to 46.22% at sgAlb target sites among adult *hF9* and *hF9*^*R338L*^ knock-in mice. Interestingly, we observed much lower indel frequencies (1.41–11.39%) in neonatal knock-in mice producing comparable levels of plasma hFIX-Padua (Fig. 6c). We speculated that the persistent expression of AAV-delivered Cas9 and sgRNAs in the adult liver may yield excessive indels at the target site, whereas rapid dilution of AAV episomes in neonatal pups reduced indel occurrence. These data revealed a previously unrecognized advantage of applying somatic gene knock-in at the neonatal stage.

A recent report of anti-Cas9 immunity in human^25,47^ raised new concerns for clinical translation of AAV-CRISPR-based gene editing therapy^48,49^. Host immunity to Cas9 was associated with potential harms, such as immune attacks to Cas9-expressing cells or induced tissue damage. Moreover, pre-existing or even post-dosing anti-Cas9 immunity might diminish gene editing outcomes by destroying the edited cells. Similar to the previous report^50^, heterogeneous anti-Cas9 activities were detected among adult mice treated with AAV-CRISPR or SpCas9 protein in our study (Fig. 7a–c, e; Supplementary Fig. 13a). Interestingly, our retro-analysis and longitudinal data supported that host anti-Cas9 activities were transient and disappeared eventually (Fig. 7c, e). Concordantly, the hFIX-Padua productions were similar in the mock and SpCas9-immunized mice after receiving AAV-KI (*hF9*^*R338L*^) injection (Fig. 7e), implicating that low-level pre-existing anti-Cas9 antibodies had negligible impact. This data was in line with Tanner et al.’s study, which showed that high-level pre-existing anti-Cas9 immunity partially negated AAV-CRISPR gene editing^48^. Altogether, these results suggested that effective gene editing would be achieved in the low anti-Cas9 background, and a potential strategy to bypass Cas9 pre-existing immunity in the clinical application can be developed through longitudinal monitoring of the anti-Cas9 activity in adult patients.

Importantly, we presented evidence that neonates had better tolerance to systemic AAV-CRISPR treatment than adult mice. AAV-KI (*hF9*) pups displayed slower immune responses and lower levels of anti-Cas9 activities (Fig. 7d, Supplementary Fig. 15a). Early exposure to SpCas9 proteins induced subtle anti-Cas9 activity and no immune memory in neonates (Fig. 7f). Markedly, low immunity to AAV2/8 was also confirmed in neonates after AAV-CRISPR treatment (Supplementary Fig. 15b), while no toxicity and adverse effect on growth and development were observed (Figs. 3a–f and 5a, b). These results suggested that administration at the neonatal stage can likely bypass the pre-existing immunity to Cas9 and AAV, which, therefore, would be more promising for gene-editing therapy.

In summary, our data demonstrated that liver-specific NHEJ knock-in through AAV-CRISPR could achieve long-term hFIX production with the lowest ever AAV dose administered for hemostasis correction. Our study addressed the key issues in developing gene-editing therapy, demonstrated the therapeutic potential of in vivo NHEJ knock-in, and explored the feasibility of clinical translation. Although more study is needed to evaluate the long-term risks associated with gene editing and to address remaining challenges, it is promising that AAV-CRISPR mediated knock-in will potentially deliver a cure for many currently unmanageable diseases.

## Methods

### Constructs

#### Cas9 and sgRNAs

AAV-nEF-SpCas9 was a gift from Juan Belmonte (Addgene plasmid #87115)^13^. AAV-LP1-SpCas9 was constructed by replacing the core EF1α (nEF) promoter in AAV-nEF-SpCas9 with LP1 promoter. sgRNAs were designed using CRISPR Design (http://crispr.mit.edu/) (Supplementary Table 1) and inserted at BbsI site in pX330 plasmid (Addgene plasmid #42230, a gift from Feng Zhang)^51^. To construct the AAV vector containing two sgRNA expression cassettes, Addgene plasmid #61591 (gift from Feng Zhang) was modified by replacing the fragment between XbaI/AflII with U6-sgA fragment from pX330 and inserting the U6-driven site-specific sgRNA between KpnI/NotI.

#### Plasmid and AAV donors

The single-cut NHEJ donor plasmid carrying ires-GFP was previously constructed^12^. scAAV vector (Addgene plasmid #21894) was modified by inserting multiple cloning sites (MCS) between Not1/Spe1. DNA fragments encoding ires-GFP-PolyA, ires-hF9-PolyA, and ires-hF9^R338L^-PolyA, with sgA target sequences at both sides, were inserted into the MCS to generate corresponding AAV NHEJ donors. To construct an AAV NHEJ donor carrying ires-Luc, nEF-Cas9 cassette in AAV-nEF-Cas9 was replaced by ires-Luc-polyA.

#### Constructs for overexpression

nEF promoter and SpCas9 in Addgene plasmid #87115 were replaced with LP1 promoter and *hF9* coding sequence to construct ssAAV-LP1-hF9. The LP1-hF9 fragment was also inserted into scAAV vector (Addgene #21894) to construct scAAV-LP1-hF9.

### AAV production

AAV packaging plasmids were obtained from Penn Vector Core at the University of Pennsylvania (UPenn), and encapsulated AAV2/8 were produced using 293FT cells (Thermo Fisher Scientific) as described previously^52^. The AAV titers (viral genome, vg) were determined using AAVpro Titration Kit (Takara Bio, #6233).

### Mice and animal experiments

Wild type ICR, C57BL/6, and *mF9−/−* (*B6.129P2-F9*^*tm1Dws*^*/J*) mice^19^ were purchased from the Jackson Laboratory (Bar Harbor, ME, USA) and maintained in the Laboratory Animal Services Centre of the Chinese University of Hong Kong. All experiments were approved by the Animal Experimentation Ethics Committee of the Chinese University of Hong Kong. During the experiment, the conditions of the animals will be monitored. Animals with any serious injury or signs of severe pain or distress will be euthanized before the end of the study.

#### Hydrodynamic injection

A total of 30 µg plasmid DNA was diluted in saline in a volume equivalent to 10% of mouse body weight and then injected into ICR mice at 6–8 weeks via their tail vein within 5–10 s. If more than one plasmid were used, 30 µg were equally divided by the number of plasmids.

#### Direct imaging for GFP expression in mouse liver

Livers dissected from sacrificed mice were saturated in PBS and directly imaged under Olympus SZX16 Stereomicroscope with a Fluorescence Imaging system.

#### AAV-CRISPR for NHEJ knock-in

AAV mixtures were prepared to consist of AAV-donor (ires-*hF9*, ires-*Luc* or ires-*hF9*^*R338L*^), AAV encoding SpCas9 (driven by either nEF or LP1 promoter), and AAV carrying sgRNAs (sgAlb/sgA or sgActb/sgA) at 2:1:1 ratio prior injections. To deliver NHEJ knock-in of *hF9* in adult *mF9−/−* mice (8–12 weeks) (Figs. 1, 4e–g), 300 µl AAV mixture containing ires-*hF9* donor, nEF- or LP1-SpCas9 and U6-sgAlb/sgA at 2:1:1 ratio was administered via tail vein at a total dose of 2 × 10^12^ vg/mouse. To knock-in of Luciferase reporter in adult ICR mice (8–12 weeks) (Fig. 4a), 300 µl AAV mixture of ires-*Luc* donor, nEF- or LP1-SpCas9 and U6-sgActb/sgA at 2:1:1 ratio was administered via tail vein at a total dose of 2 × 10^12^ vg/mouse. To deliver NHEJ knock-in of *hF9* in neonatal *mF9−/−* pups at P1–P3 (Fig. 3), 50 µl AAV mixture containing ires-*hF9* donor, nEF-SpCas9 and U6-sgAlb/sgA at 2:1:1 ratio was administered at a total dose of 4 × 10^11^ vg/mouse, either intraperitoneally (i.p.) or intravenously (i.v.) via a facial vein.

To perform dose–response analysis in Fig. 5, an AAV mixture consisting of ires-*hF9* (or ires-*hF9*^*R338L*^) donor, LP1-SpCas9 and sgAlb/sgA at 2:1:1 ratio was successively diluted to yield different doses as indicated. Fifty microlitre AAV mixtures at various doses were injected to neonatal *mF9−/−* mice (P1) via i.v. or i.p. injection, while 300 µl AAV mixtures at various doses were injected to adult *mF9−/−* mice (6–12 weeks) via i.v. injection. Donor control mice in Fig. 5c only received the ires-*hF9*^*R338L*^ donor at a dose of 4 × 10^10^ vg/mouse. Both male and female mice were used in these AAV-CRISPR-mediated NHEJ knock-in experiments.

#### AAV-based overexpression

The ssAAV or scAAV vectors carrying LP1-hF9 were injected to adult *mF9−/−* mice at 6–12 weeks via tail vein at the dose of 5 × 10^11^ vg/mouse (Fig. 1e–g). scAAV vectors carrying LP1-hF9 were i.p. injected to neonatal *mF9−/−* mice at P3 at the dose of 1 × 10^11^ vg/mouse (Fig. 1e–g). The AAV2/8 vectors carrying either nEF-SpCas9 or LP1-SpCas9 were injected to adult ICR mice at 6 weeks via tail vein at the dose of 5 × 10^11^ vg/mouse (Fig. 7a).

#### spCas9 pre-immunization

Twenty-five microgram SpCas9 protein (Invitrogen™, #A36499) was subcutaneously (s.c.) injected to adult *mF9−/−* mice (6–8 weeks). Ten microgram SpCas9 protein was i.v. or i.p. injected into neonatal *mF9−/−* mice at P1. The control mice were injected with PBS. Five weeks later, AAV mixture containing either ires-*hF9*^*R338L*^ donor, LP1-SpCas9, and sgAlb/sgA at a 2:1:1 ratio was i.v. injected into both SpCas9-immunized or PBS-treated mice, at a total input dose of 4 × 10^11^ vg/mouse.

#### Bioluminescence in vivo imaging

Mice were weighed, anesthetized using Ketamine/Xylazine (100 mg/kg and 10 mg/kg body weight respectively), and i.p injected with d-luciferin (GoldBio, #LUCK-100) at 0.15 mg/g body weight, followed by immediate bioluminescence in vivo imaging (Bruker In-vivo Xtreme Imaging System).

#### Partial hepatectomy

Mice were anesthetized using Ketamine/Xylazine (100 mg/kg and 10 mg/kg body weight, respectively) via i.p. injection and placed in the supine position. Ligation and excision of left and medium lobes were performed^53^. The excised liver tissues were fixed immediately in cold 4% paraformaldehyde or embedded in Tissue-Tek™ O.C.T. compound (Sakura Finetek™ 4583) through snap freezing in liquid nitrogen for histological analysis.

### Plasma hFIX assay

Blood specimens were collected by retro-orbital bleeding from adult mice or adolescent mice at 2–4 weeks. From pups younger than 2 weeks, blood specimens were collected by decapitation after anesthesia using Ketamine/Xylazine (100 and 10 mg/kg body weight, respectively). To collect plasma, blood specimens obtained were immediately mixed with 3.8% sodium citrate at 9:1 ratio to stop coagulation and subsequently spun down. The upper transparent plasma was then transferred into a new tube and kept at −80 °C for further use. The levels of plasma hFIX were determined by ELISA using Human Factor IX ELISA Kit (Abcam, #ab188393).

### Activated Partial thromboplastin time (aPTT) test

aPTT was measured using STart 4 Hemostasis Analyzer (Diagnostica Stago). Briefly, plasma samples collected were diluted 5 times with Owren-Koller buffer (Diagnostica Stago, #ITSG00360) and then mixed with human FIX-deficient plasma (George King Biomedical, Inc. #FIX-ID) and C.K.Prest reagent (Diagnostica Stago, #00598) at 1:1:1 ratio, followed by 37 °C incubation for 180 s. Coagulation was triggered by the addition of 25 mM calcium chloride, and clot formation time was measured by STart 4 Hemostasis Analyzer (Diagnostica Stago).

FIX activity of hFIX-Padua in Fig. 5d and Supplementary Fig. 11b was determined based on aPTT values detected using the same assay. Standard recombinant human factor IX (MonoFIX®-VF, CSL Behring) was used to generate standard curves for calculating the FIX activities. The FIX levels in the standard samples were 5 μg/ml and 50 IU/ml.

### ELISA detection of anti-SpCas9 or anti-AAV2/8 antibodies

Levels of plasma SpCas9 antibodies were measured using the ELISA protocol adopted from previous studies^25^. Briefly, SpCas9 protein (Invitrogen) was diluted in 1× coating buffer (0.2 M Na_2_CO_3_ and 0.2 M NaHCO_3_, pH 9.6) to a concentration of 2 μg/ml and coated onto 96-well ELISA plates (Corning) at 100 μl/well. Plates were incubated at 4 °C overnight and washed three times with PBS containing 0.05% Tween-20 (PBST). Plates were then blocked using 100 μl/well PBST containing 1% bovine serum albumin (BSA) (Thermo Fisher Scientific, #37525) at room temperature (RT) for 1 hour and washed three times with PBST. Mouse sera were diluted in blocking buffer, and 100 μl were added to each well in duplicate. In addition to experimental samples, a monoclonal mouse anti-SpCas9 antibody (Santa Cruz, #sc-517386) was serially diluted and added in duplicate wells to generate the standard curve. The plates were incubated at 4 °C for overnight and washed three times with PBST. Anti-mouse IgG conjugated with HRP (Cell Signaling Technology, #7076S) was diluted 1:5000 in blocking buffer and added 100 μl/well. The plates were then incubated for 1.5 h at RT and washed three times. 100 μl TMB substrate (Thermo Fisher Scientific, #N301) was added into each well and incubated for 5 min. The reaction was then stopped by adding 100 μl of stop solution (Thermo Fisher Scientific, #N600), and the optical density at 450 nm was measured using SpectraMax i3X plate reader (Molecular Devices) to determine the HRP signal. Concentrations of the plasma anti-Cas9 were normalized based on the standard curve generated using a monoclonal anti-Cas9 antibody (Santa Cruz, sc-517386).

Serum levels of AAV2/8 antibodies were measured using ELISA following a similar protocol. Briefly, AAV2/8 was diluted in 1× coating buffer to a concentration of 1 × 10^11^ vg/ml, and 100 μl/well was used to coat ELISA plates at 4 °C overnight, followed by BSA blocking for 2 h. After washing, the plasma samples were diluted at 1:10,000 and added to the plate (100 μl/well). The plates were incubated overnight at 4 °C followed by wash and subsequent incubation with HRP-conjugated anti-mouse IgG (Cell Signaling Technology, #7076S) for 1.5 h at RT. TMB substrate was then incubated for 5 min prior to signal measurement.

### Histological analysis and IHC

Freshly excised liver tissues were fixed in 4% paraformaldehyde (Sigma-Aldrich, #441244), dehydrated and embedded in paraffin, or directly snap frozen in Tissue-Tek™ O.C.T. compound (Sakura Finetek™ 4583) for cryosectioning. H&E staining was performed on 5 or 8 µm paraffin sections. Immunostaining was performed on 8 μm frozen sections. First, samples were fixed in ice-cold 4% paraformaldehyde for 15 min and washed 3 times in PBS/0.1% Triton X-100 buffer at room temperature. Then the slides were incubated with 10% normal goat serum/0.3 M glycine for 1 h to block non-specific binding, followed by overnight incubation with primary antibodies at 4 °C. After rinsing 3 times with 0.1% Triton X-100 in PBS for 15 min, samples were incubated with secondary antibodies conjugated to Alexa Fluor-488 (Invitrogen, A-11055) or Alexa Fluro 594 (Invitrogen, A-21442) at 25 °C for 2 h, followed by PBS washing. Samples were counterstained with Hoechst and finally mounted using VECTASHIELD Antifade mounting medium (Vector Laboratories, #H-1000). Primary antibodies used were rabbit polyclonal anti-hFIX/PTC (Abcam, #ab97619) at dilution of 1:200, and goat polyclonal anti-Albumin (Bethyl, #A90-134A) at dilution of 1:300. The secondary antibodies were diluted at 1:500.

### Genomic PCR analysis

Mouse genomic DNA was extracted using TIANamp Genomic DNA Kit (Tiangen, #4992254), and Phusion High-Fidelity DNA Polymerase (New England Biolabs, #M0530L) was used for each PCR reaction.

#### Indel detection via low-throughput PCR and sequencing

In silico prediction of off-target sites by sgAlb was performed using “Cas-OFFinder v2.4” (http://www.rgenome.net/cas-offinder/). Genomic fragments covering sgAlb target sequence or the top ten off-target sites were amplified from AAV-KI (*hF9*) mice. The amplicons were subjected to Sanger sequencing and the data obtained were analyzed using ICE v1.0 (Synthego) to determine indel rates^54^. The primers used were listed in Supplementary Table 3.

### RNA extraction and qRT-PCR

Total RNA was extracted from fresh or frozen tissues using TRIzol reagent (Thermo Fisher Scientific, #15596026), followed by reverse-transcription into cDNA using a High-Capacity cDNA Reverse Transcription Kit (Thermo Fisher Scientific, #4368814). PCR was then performed using the Phusion High-Fidelity DNA polymerase kit (New England Biolabs, #M0530L), and quantitative real-time RT-PCR was performed using the SYBR® Premix Ex Taq kit (Takara, #RR039A) in 7900HT Fast Real-Time PCR system (Applied Biosystems).

### RNA-seq analysis

RNA-seq libraries were constructed using VAHTS mRNA-seq V3 Library Prep Kit for Illumina (Vazyme, #NR611) and sequenced in Illumina HiSeq X by IGE BioTechnology Ltd. (Guangzhou, China). The total sequencing depths of all samples were more than 6 million in PE150 mode. RNA-seq clean reads were mapped to mouse transcript annotation of Gencode vM25 version on mm10 genome using RSEM (v1.0)^55^. The mapping rate is over 95%, and more than 5000 genes were detected from RNA-seq in all samples. The RNA-seq data were deposited into NCBI, and the accession number is PRJNA782865.

Fragments Per Kilobase Million (FPKM) values were used for the normalization and evaluation of gene expression levels. Data analysis and visualizations were performed in the R environment. Mutations and Indels were analyzed by HISAT2 (v2.2.1)^56^, HTSeq (v2.0)^57^, and SAMtools (v1.9)^58^, then were visualized and calculated in Integrative Genomics Viewer (IGV) (v2.11.4).

### Statistics and reproducibility

All data are presented as means ± SD of at least three independent experiments. Fluorescent-imaging analysis and animal studies were performed blinded and randomized. Representative experiments are shown after being repeated at least three times independently with similar results. Calculations were done using Microsoft Excel 2019, and graphs were plotted in GraphPad Prism 9 version 9.4.1. The sample sizes and *p* values are indicated in the figure graphs or the figure legends. Comparisons between the two groups were measured by two-tailed unpaired Student’s *t*-test. Differences with *p* values <0.05 were considered statistically significant.

### Reporting summary

Further information on research design is available in the Nature Portfolio Reporting Summary linked to this article.

## Supplementary information

Supplementary Information

Reporting Summary

## Data Availability

The authors confirm that the data supporting the findings in this study are available within the article and its supplementary materials. Source data are provided in this paper. The RNA-seq data generated in this study have been deposited in the NCBI Gene Expression Omnibus database under accession code PRJNA782865. Source data are provided in this paper.

## Source data

Source Data
