# Supplementary material for: Low-dose AAV-CRISPR-mediated liver-specific knock-in restored hemostasis in neonatal hemophilia B mice with subtle antibody response
Source: Nat Commun. 2022 Nov 25;13:7275. doi: 10.1038/s41467-022-34898-y (PMC9700734; doi:10.1038/s41467-022-34898-y)
Supplement: Supplementary file 1 — Supplementary Information [file 41467_2022_34898_MOESM1_ESM.pdf]

SUPPLEMENTARY INFORMATION

Low-dose AAV-CRISPR-mediated liver-specific knock-in restored hemostasis in neonatal hemophilia B mice with subtle antibody response

Xiangjun He, Zhenjie Zhang, Junyi Xue, Yaofeng Wang, Siqi Zhang, Junkang Wei, Chenzi Zhang, Jue Wang, Brian Anugerah Urip, Chun Christopher Ngan, Junjiang Sun, Yuefeng Li, Zhiqian Lu, Hui Zhao, Duanqing Pei, Chi-Kong Li, Bo Feng

SUPPLEMENTARY FIGURES:

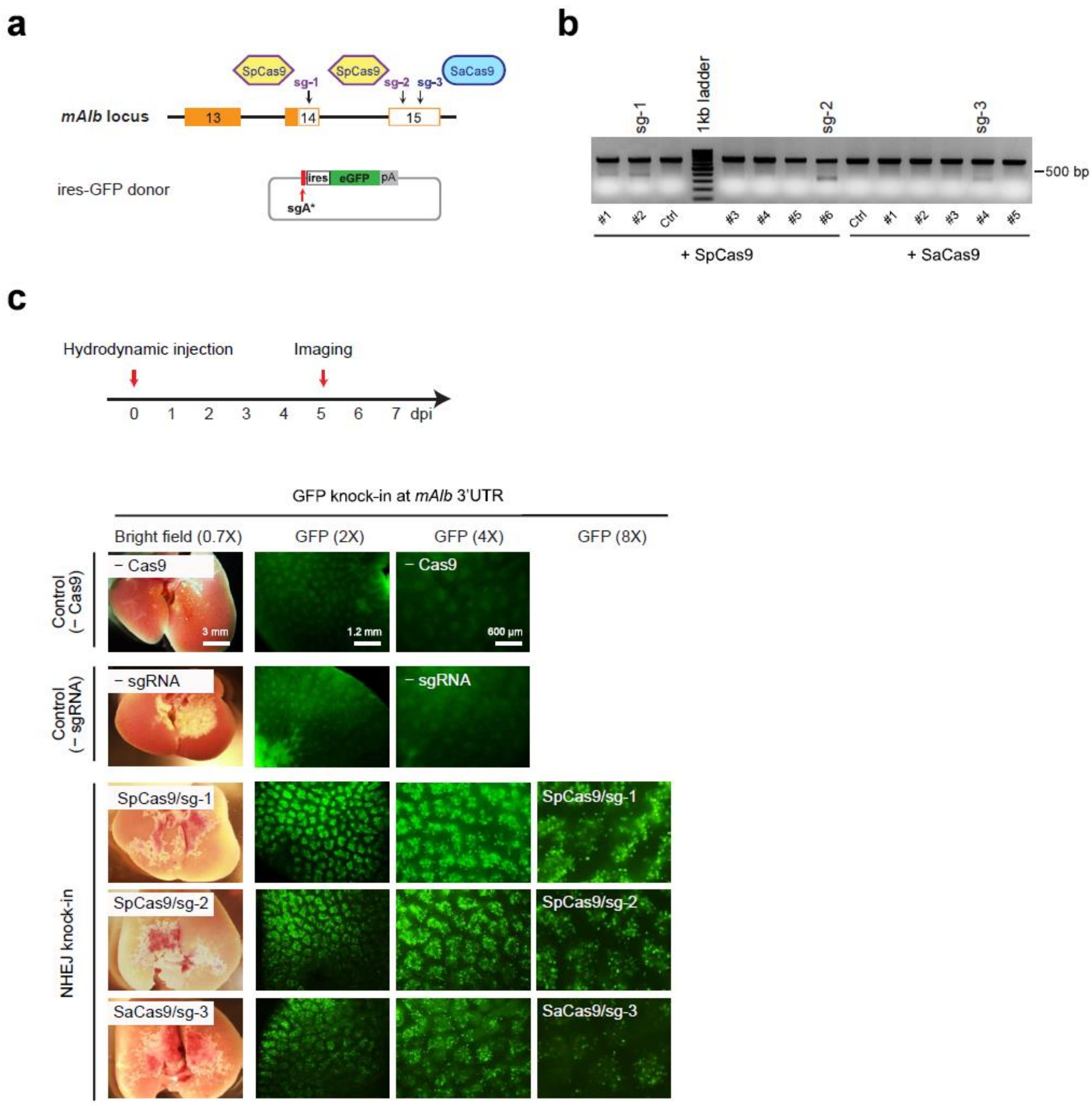

Supplementary Figure 1 | NHEJ-mediated knock-in of ires-GFP in mouse livers via hydrodynamic injection using either SpCas9 or SaCas9.

a, Schematic diagram of high-performing target sites by SpCas9/sgRNAs or SaCas9/sgRNA at *mAlb* 3'UTR and NHEJ donor plasmid carrying ires-GFP. *: sgA target sequence in donor to produce DSBs in templates for NHEJ knock-in.

b, T7E1 assay for tested SpCas9/sgRNAs or SaCas9/sgRNAs targeting at *mAlb* 3'UTR. The results of high-performing sgRNAs complexed with either SpCas9 or SaCas9 in **a** are indicated. The sequences of sgRNAs were listed in Suplementary Table 1.

c, Stereomicroscopic images of livers dissected at day 5 after hydrodynamic injection (dpi) of the plasmids in **a**.

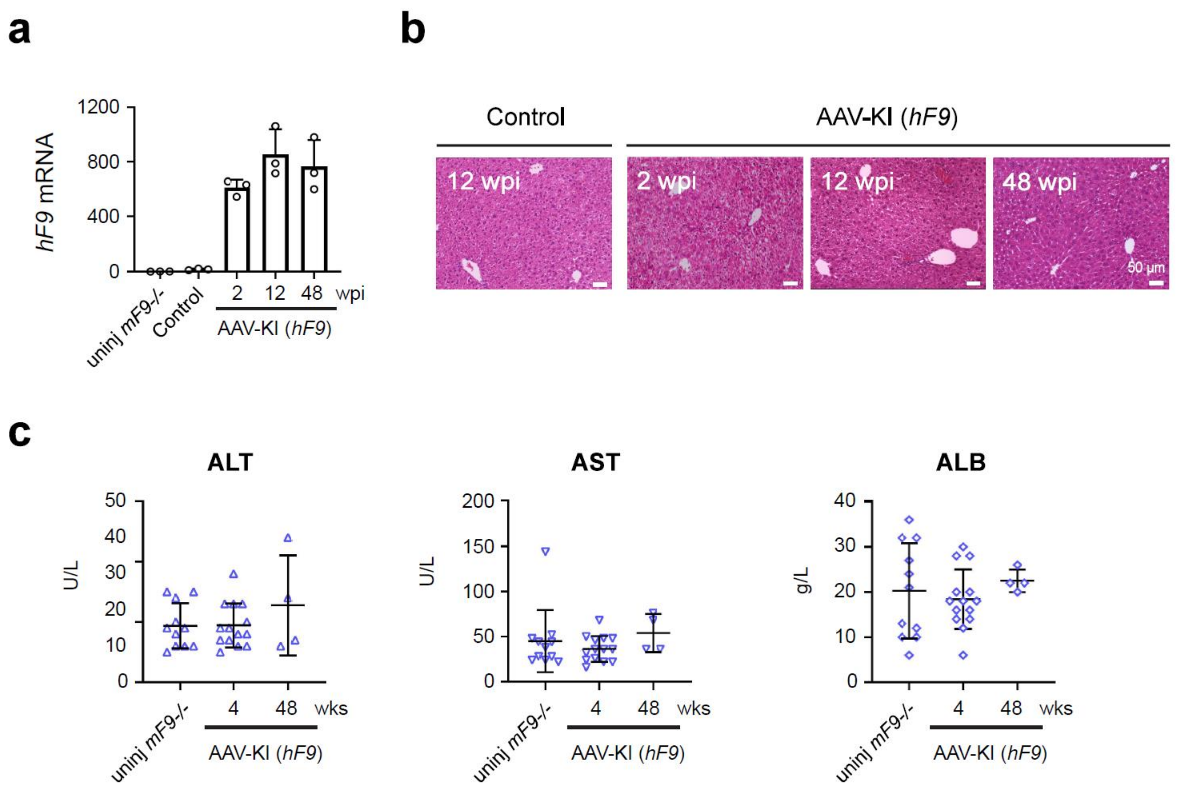

Supplementary Figure 2 | Liver function analysis in AAV-KI (*hF9*) mice.

a, Relative *hF9* mRNA levels in livers of uninjected *mF9-/-* mice, control, and *mF9-/-* mice injected with AAV2/8 carrying ires-*hF9* donor (1 × 10^{12} vg/mouse), SpCas9 (5 × 10^{11} vg/mouse) and sgRNAs (5 × 10^{11} vg/mouse). Control group was injected with ires-*hF9* donor only (1 × 10^{12} vg/mouse). qRT-PCR data shown are average ± SD (n=3).

b, H&E staining in livers from control and AAV-KI (*hF9*) mice sacrificed at 2, 12, and 48 wpi. Bar = 50 µm.

c, Levels of serum alanine aminotransferase (ALT), aspartate aminotransferase (AST) and albumin (ALB) in uninjected *mF9-/-* mice (n=11) and AAV-KI (*hF9*) mice sacrificed at 4 wpi (n=14) and 48 wpi (n=4). Data are mean ± SD.

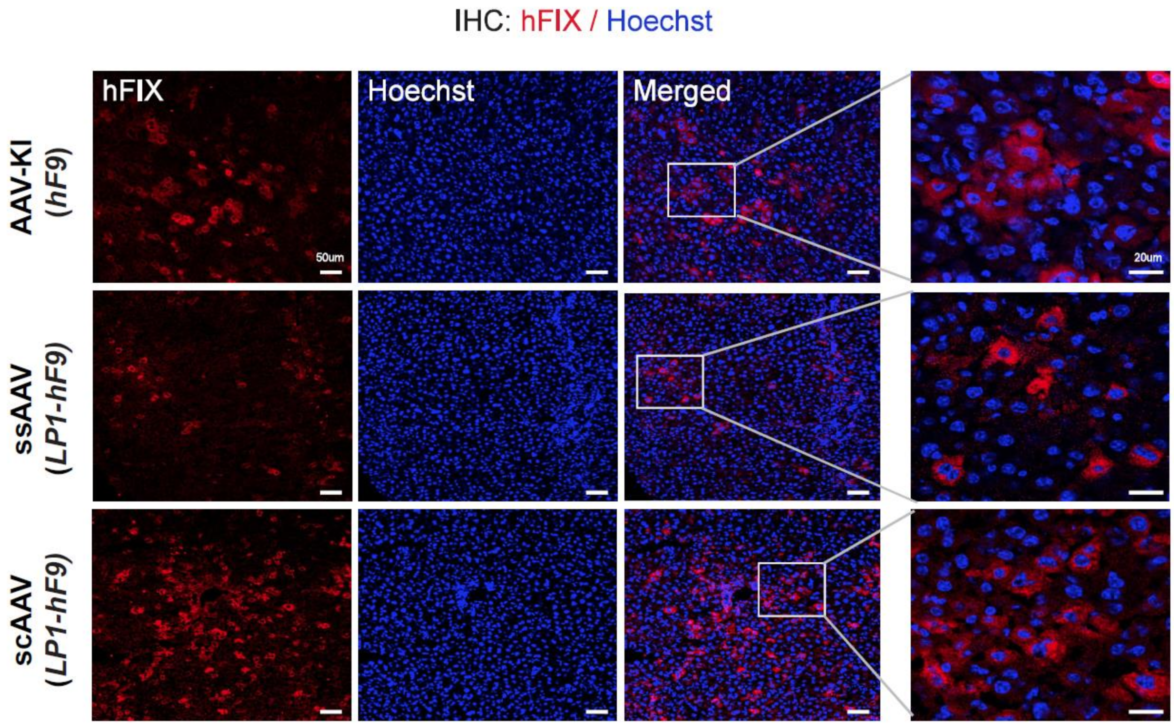

Supplementary Figure 3 | IHC staining of hFIX in livers after partial hepatectomy.
IHC staining of livers from *mF9-/-* mice treated with AAV-KI (*hF9*), ssAAV (*LP1-hF9*), or scAAV (*LP1-hF9*), followed by PHX. The mice were sacrificed at 8 weeks after PHX. Antibodies used were specific to hFIX (red). Nuclei were counterstained using Hoechst (blue). Bar = 50 µm or 20 µm (for enlarged areas).

a

RNA-seq samples for AAV-KI (*hF9*) and control mice at 2 wpi and 12 wpi

Labels	Samples	Treatments
un-1.L	uninjected mF9-/-	no treatment
un-1.R	uninjected mF9-/-	no treatment
Ctrl-1.L	Control 2wpi	AAV-ires-hF9 donor (1 ×10^12 vg/mouse)
Ctrl-1.R	Control 2wpi	AAV-ires-hF9 donor (1 ×10^12 vg/mouse)
Ctrl-2.L	Control 2wpi	AAV-ires-hF9 donor (1 ×10^12 vg/mouse)
Ctrl-2.R	Control 2wpi	AAV-ires-hF9 donor (1 ×10^12 vg/mouse)
KI-9.L	AAV-KI (hF9) 2 wpi	ires-hF9 donor (1 ×10^12) + SpCas9 (5 ×10^11) + sgRNA (5 ×10^11 vg/mouse)
KI-9.R	AAV-KI (hF9) 2 wpi	ires-hF9 donor (1 ×10^12) + SpCas9 (5 ×10^11) + sgRNA (5 ×10^11 vg/mouse)
KI-10.L	AAV-KI (hF9) 2 wpi	ires-hF9 donor (1 ×10^12) + SpCas9 (5 ×10^11) + sgRNA (5 ×10^11 vg/mouse)
KI-10.R	AAV-KI (hF9) 2 wpi	ires-hF9 donor (1 ×10^12) + SpCas9 (5 ×10^11) + sgRNA (5 ×10^11 vg/mouse)
Ctrl-3.L	Control 12 wpi	AAV-ires-hF9 donor (1 ×10^12 vg/mouse)
Ctrl-3.R	Control 12 wpi	AAV-ires-hF9 donor (1 ×10^12 vg/mouse)
Ctrl-F4.L	Control 12 wpi	AAV-ires-hF9 donor (1 ×10^12 vg/mouse)
Ctrl-F4.R	Control 12 wpi	AAV-ires-hF9 donor (1 ×10^12 vg/mouse)
KI-1.L	AAV-KI (hF9) 12 wpi	ires-hF9 donor (1 ×10^12) + SpCas9 (5 ×10^11) + sgRNA (5 ×10^11 vg/mouse)
KI-1.R	AAV-KI (hF9) 12 wpi	ires-hF9 donor (1 ×10^12) + SpCas9 (5 ×10^11) + sgRNA (5 ×10^11 vg/mouse)
KI-2.L	AAV-KI (hF9) 12 wpi	ires-hF9 donor (1 ×10^12) + SpCas9 (5 ×10^11) + sgRNA (5 ×10^11 vg/mouse)
KI-2.R	AAV-KI (hF9) 12 wpi	ires-hF9 donor (1 ×10^12) + SpCas9 (5 ×10^11) + sgRNA (5 ×10^11 vg/mouse)

b

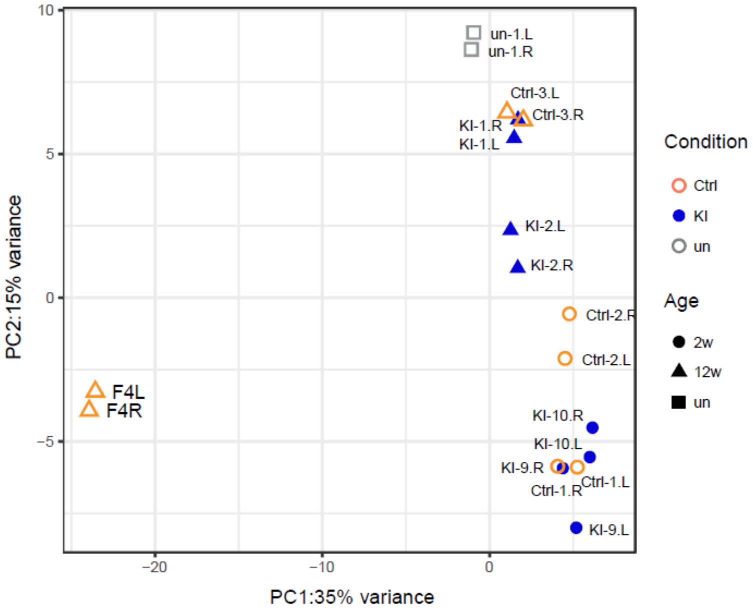

Supplementary Figure 4 | RNA-seq analysis of liver samples from AAV-KI (*hF9*) and control mice at 2 wpi and 12 wpi.
a, List of RNA-seq samples and treatment performed in each mice.
b, Principal component analysis (PCA) based on the normalized RNA-seq data.

a

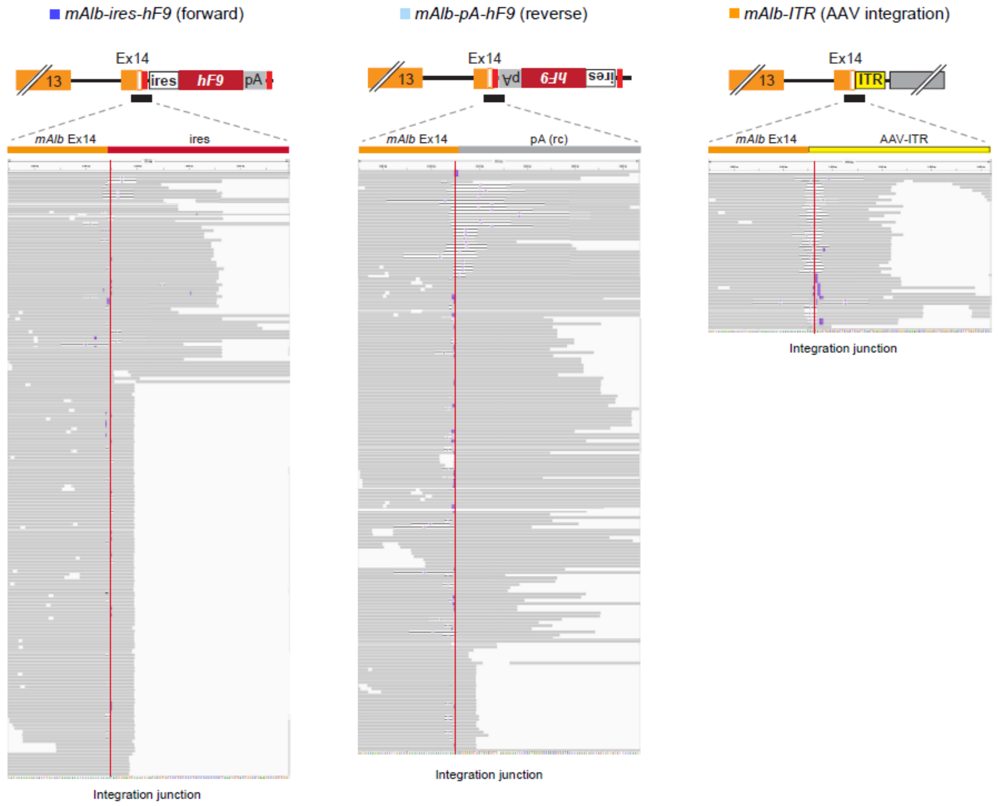

b

Group	Samples	*mAlb-ires-hF9* (forward insertion)		*mAlb-pA-hF9* (reverse insertion)		*mAlb-ITR* (AAV integration)	
Uninj	un-1.L	0.00%	0.00%	0.00%	0.00%	0.00%	0.00%
	un-1.R	0.00%		0.00%		0.00%	
Ctrl_2w	Ctrl-1.L	0.00%	0.00%	0.00%	0.00%	0.00%	0.00%
	Ctrl-1.R	0.00%		0.00%		0.00%	
	Ctrl-2.L	0.00%		0.00%		0.00%	
	Ctrl-2.R	0.00%		0.00%		0.00%	
KI_2w	KI-10.L	1.24%	1.04 ± 0.14%	1.36%	1.41 ± 0.15%	0.26%	0.38 ± 0.14%
	KI-10.R	1.10%		1.20%		0.25%	
	KI-9.L	0.95%		1.49%		0.59%	
	KI-9.R	0.86%		1.60%		0.41%	
Ctrl_12w	Ctrl-3.L	0.00%	0.00%	0.00%	0.00%	0.00%	0.00%
	Ctrl-3.R	0.00%		0.00%		0.00%	
	Ctrl-F4.L	0.00%		0.00%		0.00%	
	Ctrl-F4.R	0.00%		0.00%		0.00%	
KI_12w	KI-1.L	3.22%	3.26 ± 0.09%	3.93%	5.25 ± 0.81%	0.83%	0.72 ± 0.12%
	KI-1.R	3.22%		5.33%		0.73%	
	KI-2.L	3.17%		5.62%		0.80%	
	KI-2.R	3.41%		6.12%		0.51%	

Supplementary Figure 5 | Chimeric transcripts detected by RNA-seq analysis in AAV-KI (*hF9*) mice.
a, Modifications at *mAlb* locus corresponding to three chimeric transcripts analyzed in **Fig. 2e**, and alignments between junction templates and RNA reads. The schematic diagrams (upper panels) showed modified *mAlb* locus with 1) targeted insertions of ires-*hF9* at either forward or 2) reverse orientation, or 3) integration of AAV vector at the sgAlb target site. The black bars indicated junction regions examined in subsequent alignments. Representative alignment data (lower panels) from AAV-KI (*hF9*) sample KI-2.L were shown. Red lines indicated the integration junctions. rc: reverse complementary.
b, Percentages of chimeric transcripts in **a.** among total *mAlb*-derived transcripts. Data were calculated using Integrative Genomics Viewer (IGV), based on the RNA-reads mapped to corresponding integration junctions.

Tools for *in silico* off-target prediction

No.	Tools	Webpages
1	Benchling	https://www.benchling.com/crispr/
2	Cas-OFFinder	http://www.rgenome.net/cas-offinder/
3	CasOT	http://eendb.zfgenetics.org/casot/
4	CCTop	https://cctop.cos.uni-heidelberg.de:8043/
5	CFD/CRISPick	https://portals.broadinstitute.org/gppx/crispick/public
6	CHOPCHOP	https://chopchop.cbu.uib.no/
7	CrisFlash	https://github.com/crisflash
8	CRISPOR	http://crispor.tefor.net/
9	CRISPR-DT	http://bioinfolab.miamioh.edu/CRISPR-DT/
10	CRISPR-GE	http://skl.scau.edu.cn/
11	CRISPR-Plant v2	http://www.genome.arizona.edu/crispr2/
12	CRISTA	https://crista.tau.ac.il/download.html
13	CROP	https://github.com/vaprilyanto/crop
14	CROP-IT	http://www.adlilab.org/CROP-IT/homepage.html
15	CT-Finder	http://bioinfolab.miamioh.edu/ct-finder/
16	DeepCRISPR	http://www.deepcrispr.net/
17	Deskgen	https://www.deskgen.com/landing/cloud.html
18	E-CRISP	http://www.e-crisp.org/E-CRISP/designcrispr.html
19	Elevation	https://crispr.ml/
20	OfftPredict	https://github.com/penn-hui/OfftargetPredict
21	FlashFry	https://mckennalab.github.io/FlashFry/
22	flyCRISPR	http://targetfinder.flycrispr.neuro.brown.edu/
23	Guide-Design	https://zlab.bio/guide-design-resources
24	MIT	http://www.genome-engineering.org/
25	OffScan	https://bmcgenomics.biomedcentral.com/articles/10.1186/s12864-019-6241-9
26	Off-Spotter	https://cm.jefferson.edu/Off-Spotter/
27	Synthego	https://www.synthego.com/products/bioinformatics/crispr-design-tool
28	TrueDesign	https://www.thermofisher.com/hk/en/home/life-science/genome-editing/geneart-crispr/invitrogen-truedesign-genome-editor.html

Supplementary Figure 6 | Summary of *in silico* off-target prediction tools.

a

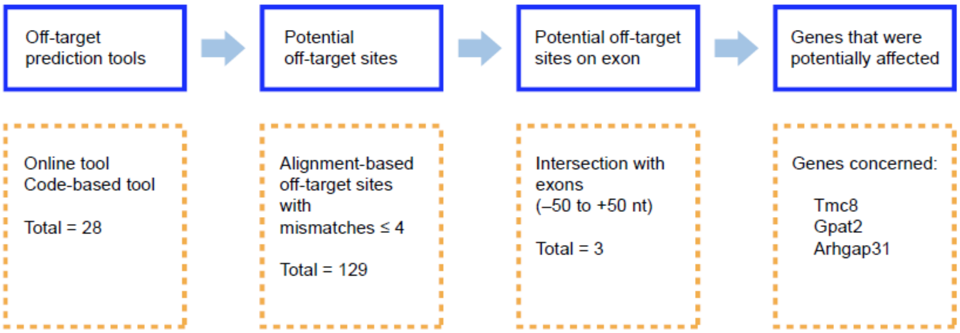

b

Exonic off-target sites

Target site	DNA sequnece	PAM	Chromosome	Position	Direction	Gene	Mismatches
sgAlb target site	ATAGTTACCTGAGAAGGTTG	TGG	chr5	90475321	-	Alb	0
Off-target ex 1	ATAGTCACCTGAGCAGAATG	AGG	chr11	117791587	-	Tmc8	4
Off-target ex 2	GTAGTCACCTGAGGAGGTTT	GGG	chr16	38624539	+	Arhgap31	4
Off-target ex 3	ATAGTTACCTGAGCAAGAAG	GGG	chr2	127429111	-	Gpat2	4

c

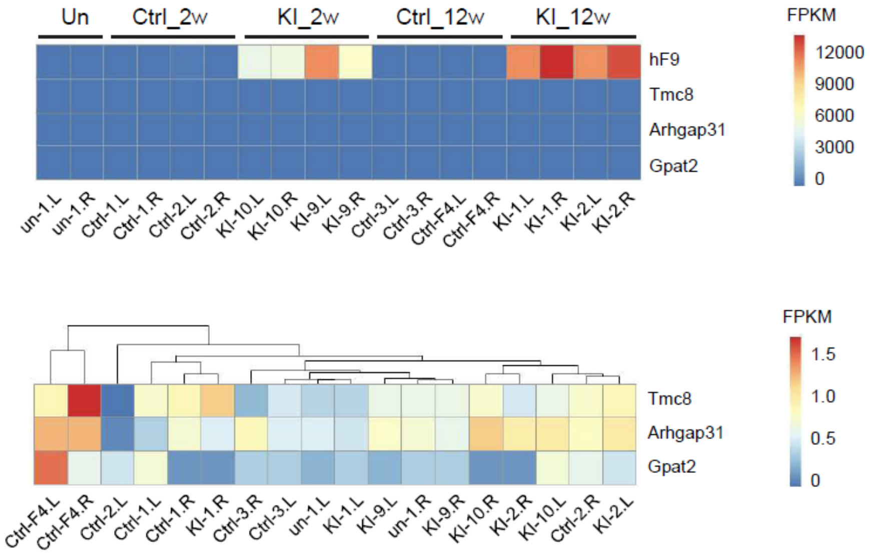

Supplementary Figure 7 | *In silico* prediction of exonic off-target sequences of sgAlb.
a, The pipeline and results of *in silico* prediction.
b, Summary of three candidate sgAlb off-target sequences located in exons.
c, Expression heat maps of corresponding genes in **b,** compared to *hF9* transgene (Upper) or clustered based on their RNA-Seq data (Lower).

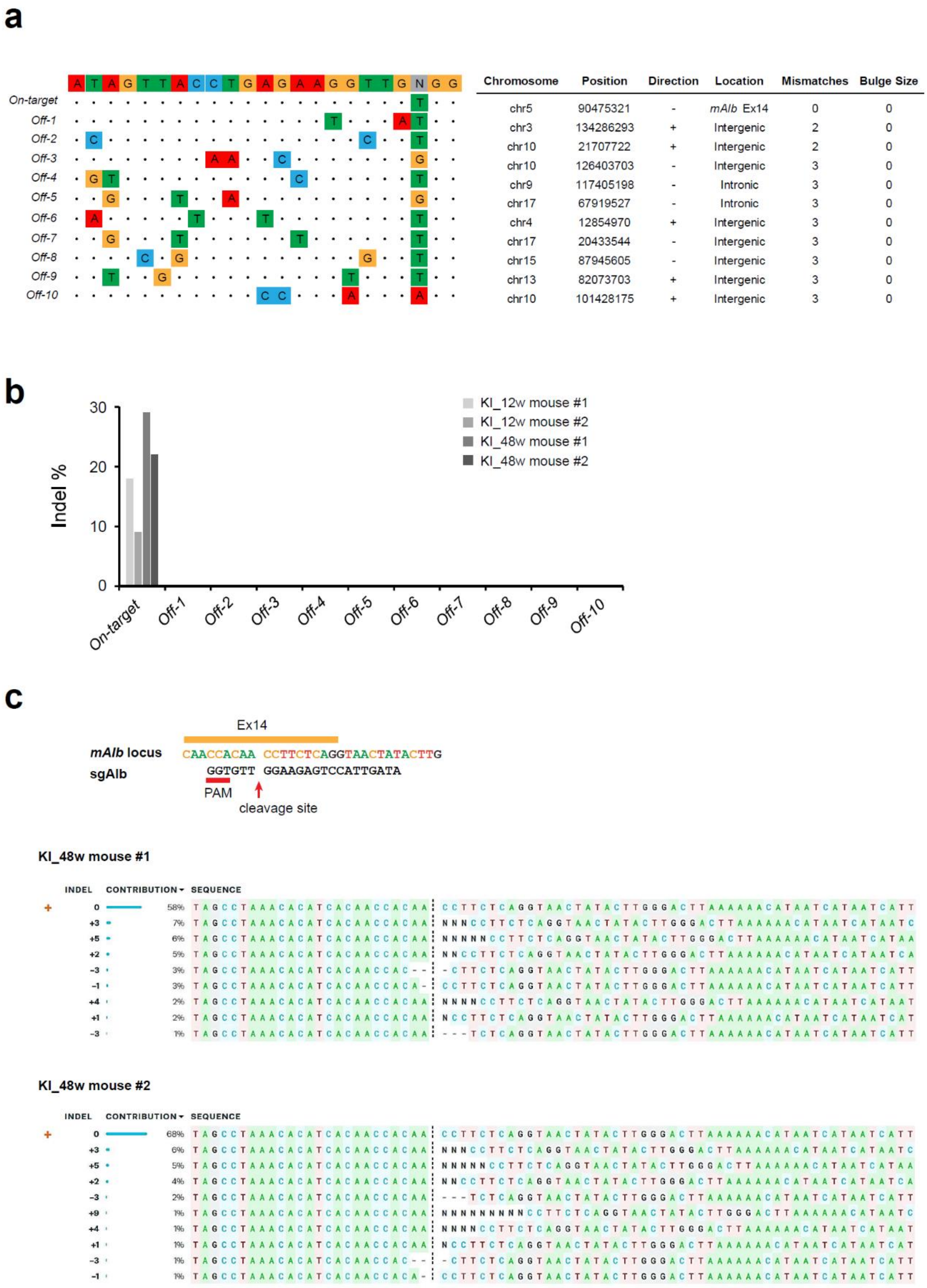

Supplementary Figure 8 | Low-throughput PCR-seq analysis of candidate off-target sites in AAV-KI (*hF9*) mouse genome.
a, sgAlb target sequence and top 10 off-target candidate sequences predicted using Cas-OFFinder in mouse genome (mm10).
b, Rates of indels calculated in liver genome of AAV-KI (*hF9*) mice (n=4) by low-throughput PCR-seq followed by ICE analysis.
c, Representative ICE data from two AAV-KI (*hF9*) liver DNAs collected at 48 wpi.

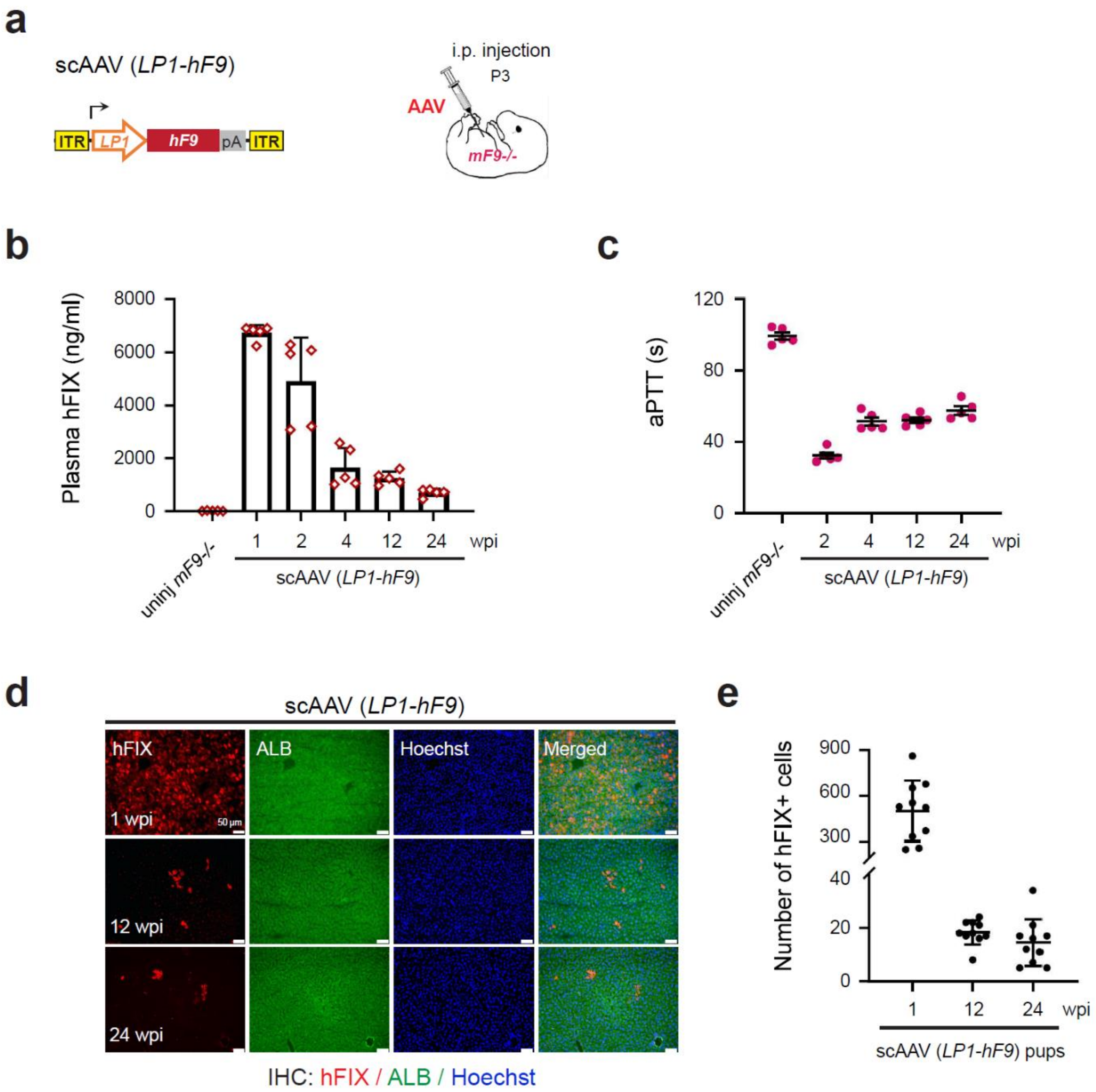

Supplementary Figure 9 | Episomal *hF9* expression using scAAV vector in neonatal hemophilia B mice.

a, Schematic diagram of scAAV vector carrying *LP1-hF9* and injection method (i.p.) in neonatal *mF9-/-* pups at P3.

b, Plasma hFIX levels in neonatal *mF9-/-* mice after receiving i.p. injection of scAAV (*LP1-hF9)* at P3. Plasma from uninjected *mF9-/-* pups were used as control. Data are mean ± SD (n=5).

c, aPTT values in plasma samples from mice in **b**. Data are mean ± SD (n=5).

d, IHC staining of livers from *mF9-/-* pups treated with scAAV (*LP1-hF9)* at P3 and sacrificed at 1,12, and 24 wpi. Antibodies used were specific to hFIX (red) and endogenous mouse ALB (green). Nuclei were counterstained using Hoechst (blue). Bar = 50 µm.

e, Quantitation of hFIX-positive hepatocytes in **d**. hFIX-positive cells were counted from 10 randomly selected fields. Data are mean ± SD.

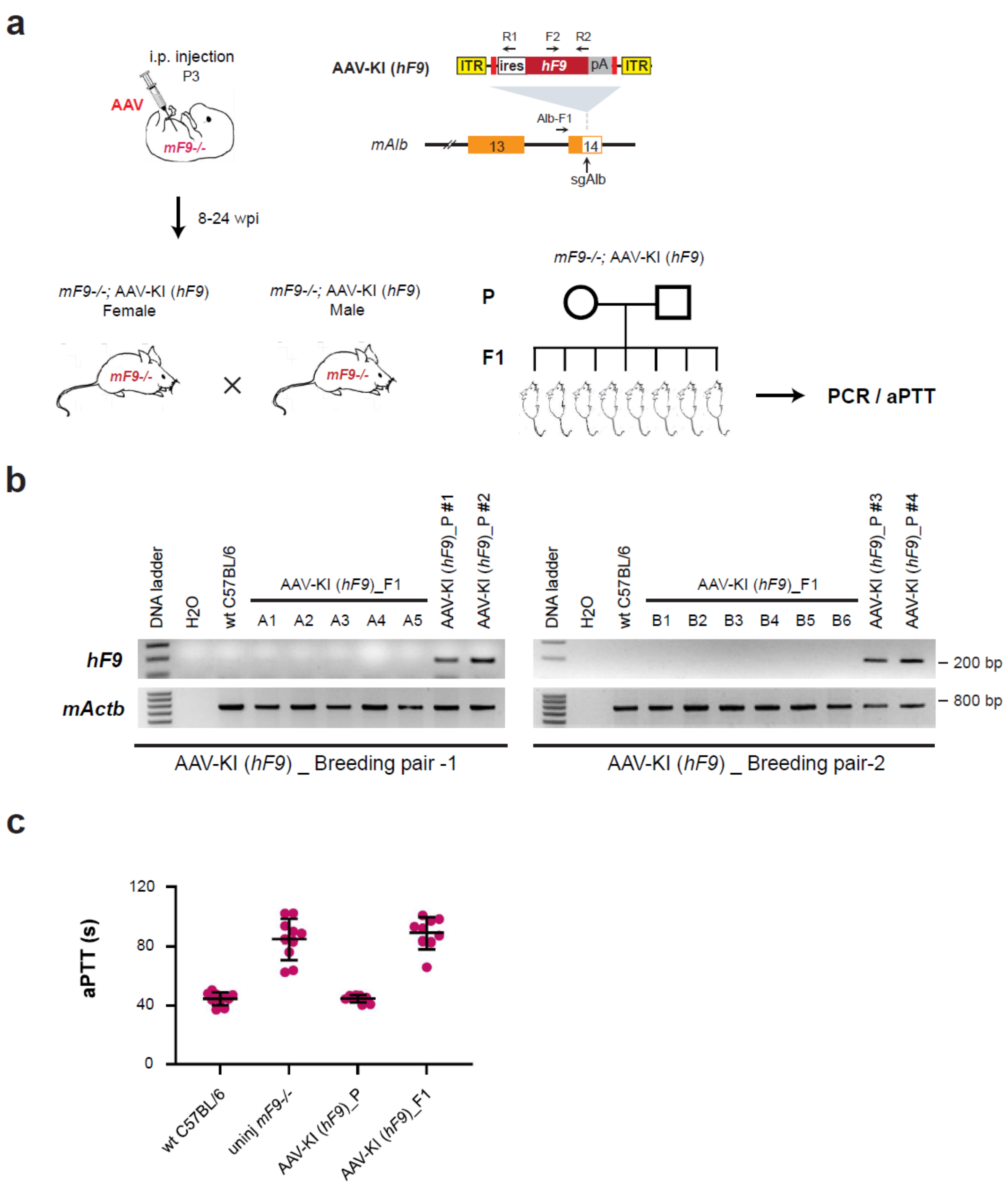

Supplementary Figure 10 | Offspring of AAV-KI (*hF9*) pups did not inherit *hF9* integration.
a, Schematics for AAV-CRISPR mediated *hF9* knock-in in *mF9-/-* neonates. They were bred during 8-24 wpi, and their offspring (AAV-KI (*hF9*)_F1) was analyzed by genome PCR and plasma assay.
b, Detection of targeted insertion of ires-*hF9* at *mAlb* 3'UTR. Liver genome DNA collected from two AAV-KI (*hF9*) breeding pairs and their offspring were analyzed by genome PCR. Primer sequences for detecting *hF9* integration (F2/R2) were listed in **Supplementary Table 2.** Primer binding sites (F2/R2) were indicated in **a**.
c, aPTT values in plasma samples from wt C57BL/6, uninjected *mF9-/-*, AAV-KI (*hF9*) pups, and AAV-KI (*hF9*)_F1. Each data point represents an individual mouse. Data are mean ± SD (n=10).

a

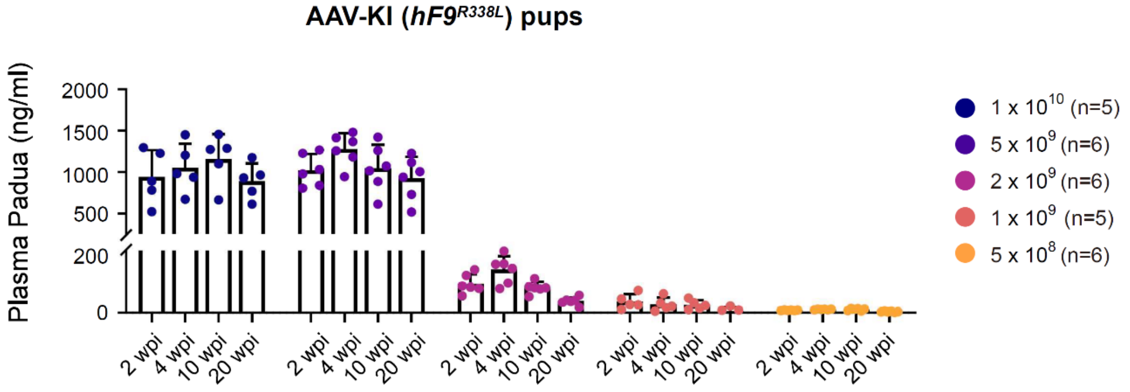

b

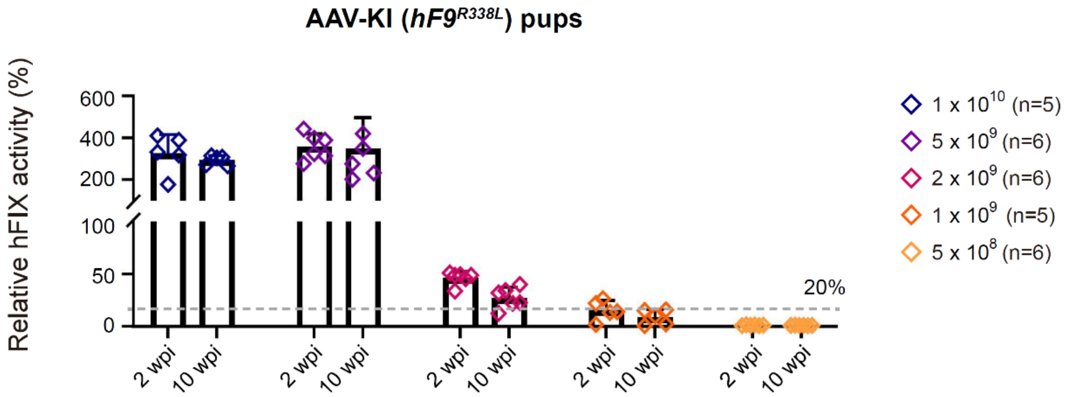

Supplementary Figure 11 | Plasma hFIX-Padua levels and relative hFIX activities in AAV-KI (*hF9^{R388L}*) pups receiving various AAV doses through i.v. injection over 20-week observation period.

a, Plasma hFIX-Padua levels of i.v. injected AAV-KI (*hF9^{R388L}*) pups in **Fig. 5b** at 2, 4, 10 and 20 wpi. Data are mean ± SD. Number of mice per group (n) was indicated in the bracket after the dose.

b, Relative hFIX activities in blood samples collected at 2 wpi and 10 wpi in **a**. Total AAV doses (vg/mouse) used in each group were indicated in the legends and the group sizes were indicated in brackets. The standard curve was generated using recombinant human factor IX (MonoFIX) to calculate relative hFIX activity. Data are mean ± SD. Number of mice per group (n) was indicated in the bracket after the dose.

RNA-seq samples for adult and neonatal AAV-KI (*hF9*^{R338L}) mice receiving various AAV input doses

Labels	Time point	Treatment	Total AAV input
A_dose-1	7 mpi	ires-hF9^{R338L} donor (1 × 10^12) + SpCas9 (5 × 10^11) + sgRNA (5 × 10^11)	2 × 10^12 vg/mouse
A_dose-2	7 mpi	ires-hF9^{R338L} donor (2 × 10^11) + SpCas9 (1 × 10^11) + sgRNA (1 × 10^11)	4 × 10^11 vg/mouse
A_dose-3.1	7 mpi	ires-hF9^{R338L} donor (4 × 10^10) + SpCas9 (2 × 10^10) + sgRNA (2 × 10^10)	8 × 10^10 vg/mouse
A_dose-3.2	6 mpi	ires-hF9^{R338L} donor (4 × 10^10) + SpCas9 (2 × 10^10) + sgRNA (2 × 10^10)	8 × 10^10 vg/mouse
A_dose-3.3	6 mpi	ires-hF9^{R338L} donor (4 × 10^10) + SpCas9 (2 × 10^10) + sgRNA (2 × 10^10)	8 × 10^10 vg/mouse
A_dose-4	6 mpi	ires-hF9^{R338L} donor (2 × 10^10) + SpCas9 (1 × 10^10) + sgRNA (1 × 10^10)	4 × 10^10 vg/mouse
A_dose-5	6 mpi	ires-hF9^{R338L} donor (1 × 10^10) + SpCas9 (5 × 10^9) + sgRNA (5 × 10^9)	2 × 10^10 vg/mouse
A_dose-6	6 mpi	ires-hF9^{R338L} donor (5 × 10^9) + SpCas9 (2.5 × 10^9) + sgRNA (2.5 × 10^9)	1 × 10^10 vg/mouse
P_dose-1	6 mpi	ires-hF9^{R338L} donor (5 × 10^9) + SpCas9 (2.5 × 10^9) + sgRNA (2.5 × 10^9)	1 × 10^10 vg/mouse
P_dose-2.1	6 mpi	ires-hF9^{R338L} donor (2.5 × 10^9) + SpCas9 (1.25 × 10^9) + sgRNA (1.25 × 10^9)	5 × 10^9 vg/mouse
P_dose-2.2	6 mpi	ires-hF9^{R338L} donor (2.5 × 10^9) + SpCas9 (1.25 × 10^9) + sgRNA (1.25 × 10^9)	5 × 10^9 vg/mouse
P_dose-3	6 mpi	ires-hF9^{R338L} donor (1 × 10^9) + SpCas9 (5 × 10^8) + sgRNA (5 × 10^8)	2 × 10^9 vg/mouse
P_dose-4	6 mpi	ires-hF9^{R338L} donor (5 × 10^8) + SpCas9 (2.5 × 10^8) + sgRNA (2.5 × 10^8)	1 × 10^9 vg/mouse
P_dose-5.1	6 mpi	ires-hF9^{R338L} donor (2.5 × 10^8) + SpCas9 (1.25 × 10^8) + sgRNA (1.25 × 10^8)	5 × 10^8 vg/mouse
P_dose-5.2	6 mpi	ires-hF9^{R338L} donor (2.5 × 10^8) + SpCas9 (1.25 × 10^8) + sgRNA (1.25 × 10^8)	5 × 10^8 vg/mouse

Supplementary Figure 12 | RNA-seq samples for adult and neonatal AAV-KI (*hF9*^{R338L}) receiving various AAV input doses.

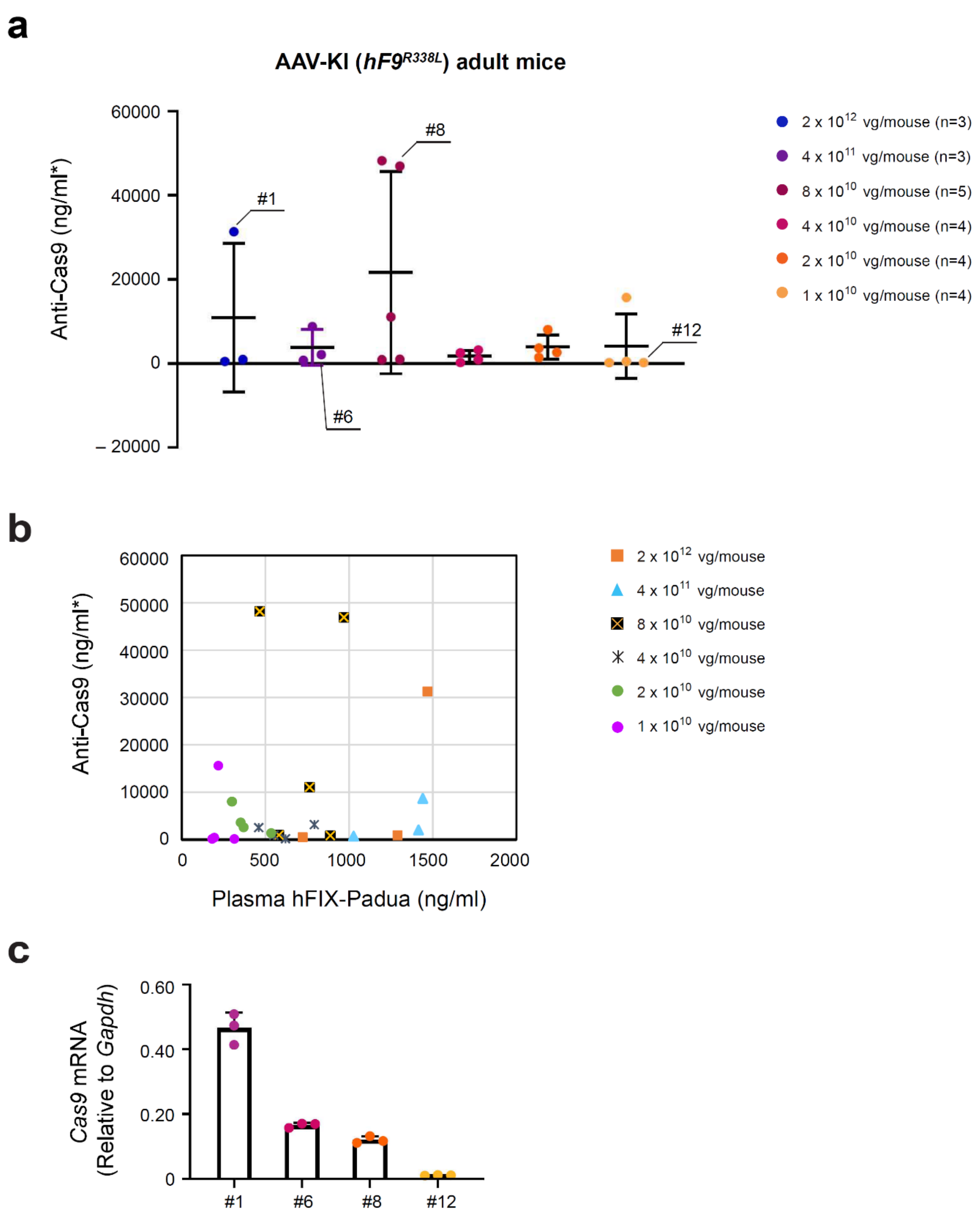

Supplementary Figure 13 | Levels of anti-Cas9 antibodies in AAV-KI (*hF9*^{R338L}) mice receiving different AAV doses.

a, Levels of plasma anti-Cas9 antibodies in AAV-KI (*hF9*^{R338L}) mice receiving different AAV doses at 6 wpi (**Fig. 5c).** Each dot represents one mouse. Ctrl: control mice injected with AAV-donor only. Data are mean ± SD. *Concentrations were normalized based on the standard curve generated using monoclonal anti-Cas9 antibody (sc-517386). Liver RNAs extracted from mice #1, #6, #8 and #12 were selected for RT-PCR analysis in **c**. Number of mice per group (n) was indicated in the bracket after the dose.

b, Scatter plot of anti-Cas9 activities and plasma hFIX-Padua levels in mice indicated in **a**. Each dot represents one mouse and dose groups were indicated by different colors.

c, Relative *SpCas9* mRNA levels in liver of mice indicated in **a.** qRT-PCR data shown are average ± SD (n=3).

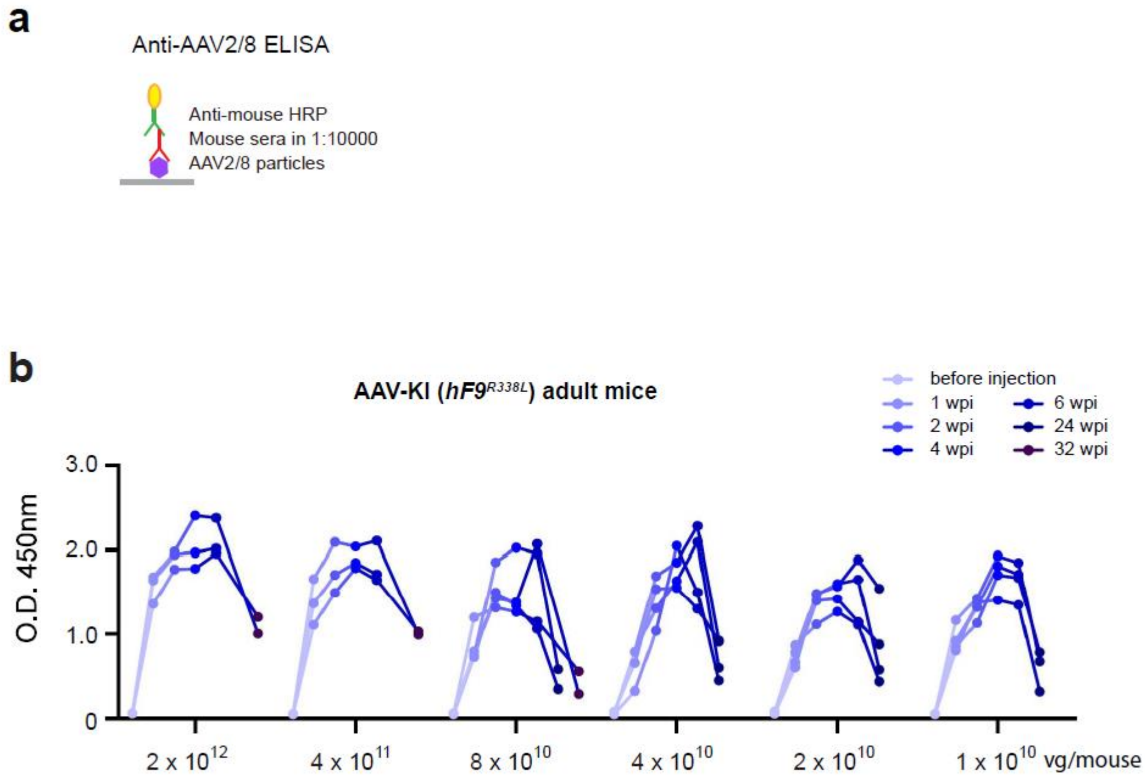

Supplementary Figure 14 | ELISA detection of plasma anti-AAV2/8 antibodies in adult AAV-KI (Padua) mice receiving different AAV doses.
a, Schematics of ELISA assay for plasma anti-AAV2/8 antibody detection.
b, ELISA detection of plasma anti-AAV2/8 antibodies in mice receiving different AAV doses (**Fig. 5c**) at 1, 2, 3, and 4 wpi. plasma samples were diluted to 1:10000. Each dot represents one mouse. Samples collected at different time points were indicated by different colors.

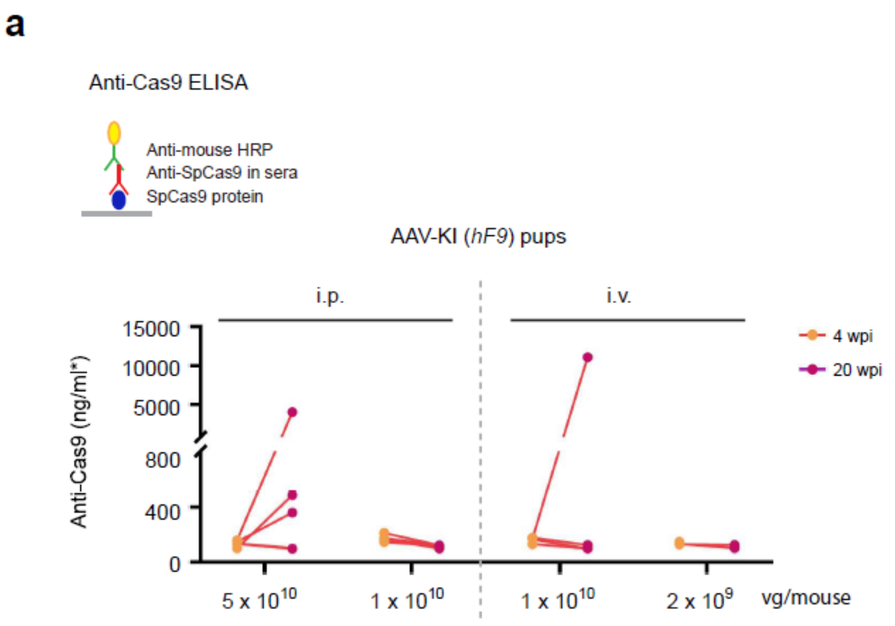

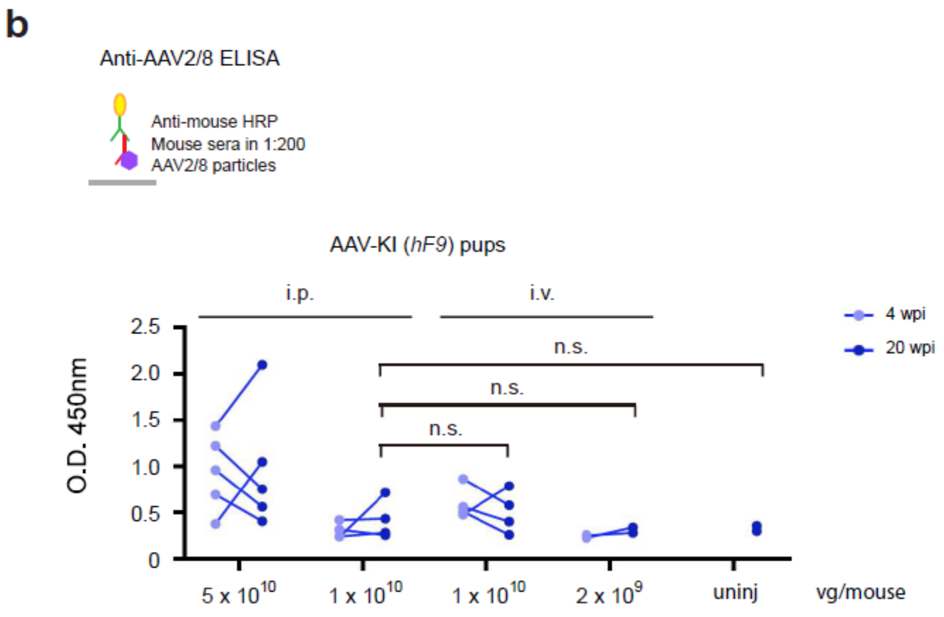

Supplementary Figure 15 | Plasma anti-Cas9 and anti-AAV2/8 antibodies in AAV-KI (*hF9*) pups receiving low AAV doses through i.v. and i.p. injections.
a, Levels of plasma anti-Cas9 antibody detected by ELISA in the AAV-KI (*hF9*) pups receiving low AAV doses through i.v. and i.p. injections in **Figure 5a.** Each dot represents one mouse. Data at different time points were indicated in different colors. *Concentrations were normalized based on the standard curve generated using monoclonal anti-Cas9 antibody (sc-517386).
b, ELISA detection of plasma anti-AAV2/8 antibodies in **a.** Plasma samples were diluted in 1:200 ratio. Each dot represents one mouse. Data at different time points were indicated in different colors. Statistical analysis was performed using two-tailed unpaired T-test. n.s., not significant.

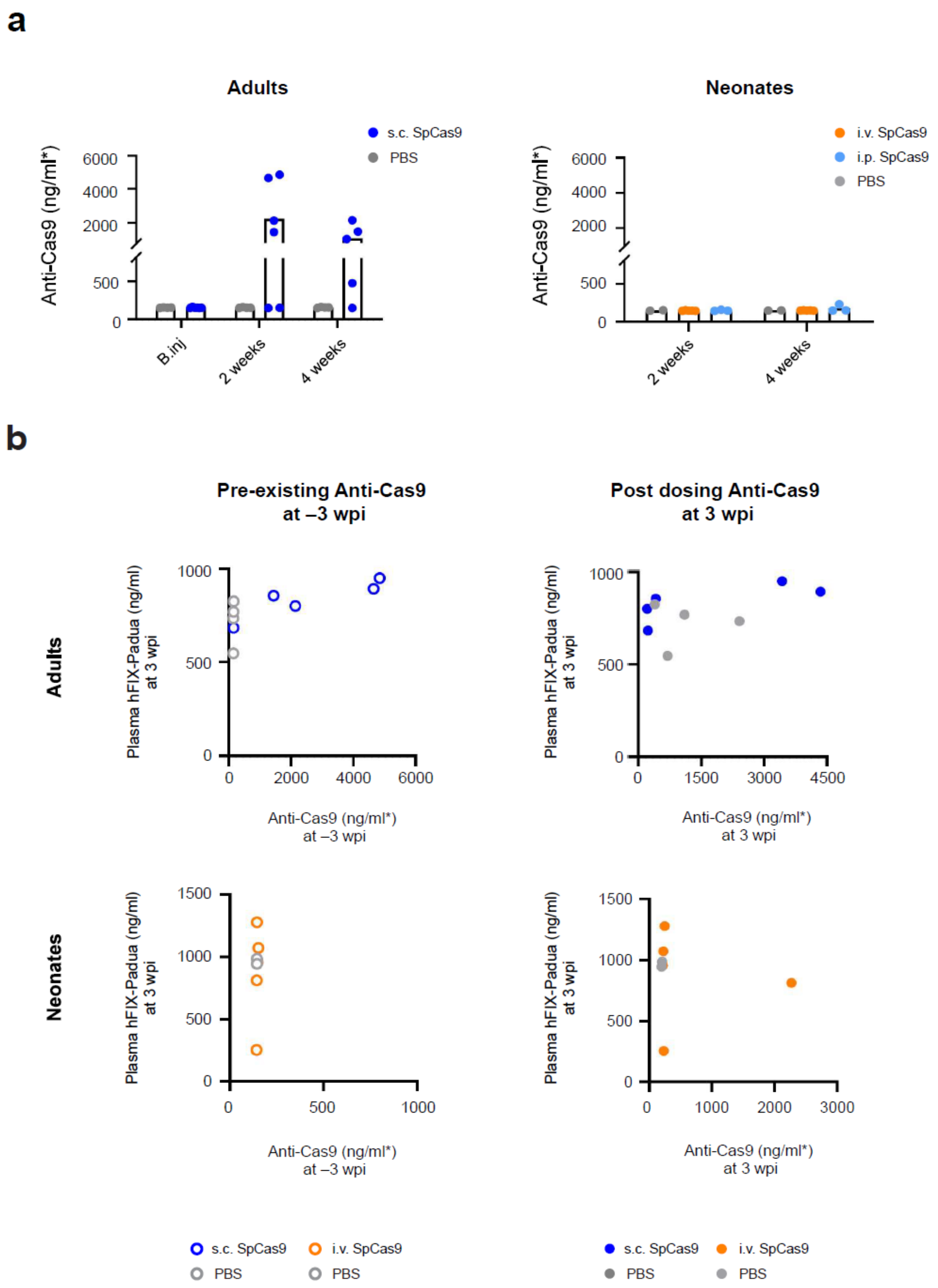

Supplementary Figure 16 | Plasma hFIX-Padua and anti-Cas9 activities in SpCas9-immunized mice before and after receiving AAV-KI (*hF9*^{R338L}).

a, Levels of plasma anti-Cas9 antibody in adult and neonatal *mF9-/-* mice after immunization with SpCas9 protein. Adult *mF9-/-* mice were s.c. injected with 25 µg SpCas9 protein, while neonatal *mF9-/-* mice were treated with 10 µg SpCas9 protein via either i.v. or i.p. injections. Plasma assay results from samples collected at 2 weeks and 4 weeks post immunization were shown. Each dot represented the measurement from a single mouse. Data are mean ± SD (n=5).

b, Scatter plots of anti-Cas9 activities and plasma hFIX-Padua levels in adult (upper panels) and neonatal (lower panels) pre-immunized mice before and after receiving AAV-KI (*hF9*^{R338L}) in **Fig. 7e, f**. Data at -3 wpi and 3 wpi from AAV administration were shown. Each dot represents one mouse. Pre-immunized mice and control groups were indicated in different colors.

Supplementary Table 1. sgRNA sequences tested.

sgRNA	Name	Sequence	PAM	Target location	Number of off-target sites		
					1 mismatch	2 mismatch	3 mismatch
SpCas9 #1	sgAlb/sg-1	ATCACAACCACAACCTTCTC	AGG	*mAlb* Ex14	0	0	16
SpCas9 #2		ATAGTTACCTGAGAAGGTTG	TGG	*mAlb* Ex14	0	2	12
SpCas9 #3		TCAGGACTCATCTTTTCTGT	TGG	*mAlb* Ex15	0	1	21
SpCas9 #4		GTGCTGCAATTAATAAAAAA	TGG	*mAlb* Ex15	0	0	25
SpCas9 #5		TAAAGAAATTTGTGTTCCTT	AGG	*mAlb* Ex15	0	2	29
SpCas9 #6	sg-2	GGTGTAAAATCAACACCCTA	AGG	*mAlb* Ex15	0	0	4
SaCas9 #1		CTGAGTCTTCATGTCTTTTTTTCT	CAGGGT	*mAlb* Ex15	0	0	0
SaCas9 #2		TTACACCAACAGAAAAGATGAGTC	CTGAGT	*mAlb* Ex15	0	0	0
SaCas9 #3		GTTGATTTTACACCAACAGAAAAG	ATGAGT	*mAlb* Ex15	0	0	0
SaCas9 #4		ATGTTTAAAGAAATTTGTGTTCCT	TAGGGT	*mAlb* Ex15	0	0	0
SaCas9 #5	sg-3	GTGCTGCAATTAATAAAAAATGGA	AAGAAT	*mAlb* Ex15	0	0	0
sgActb	sgActb	GCCCCAAAGTTCTACAAATG	TGG	*mActb* Ex6	0	0	9

Supplementary Table 2. Primers used for PCR.

Primers	Sequences
Genome PCR for integration detection	
Alb-F1	ACTTGCAAAGCCTAGAATCATGAAC
R1	CCTCACATTGCCAAAAGACG
F2	TTCACAAACTTCTAAGCTCACCCGTGCTG
R2	AAAACAACCTGCCAAGGGAATTGACCTG
mActb-F1	TGTTACTGAGCTGCGTTTTACACC
mActb-R1	CCTAGCCCACCCTAGATGCC
qRT-PCR	
Gapdh	GGGCATCTTGGGCTACACTGAGGACCAG<br>CACCCTGTTGCTGTAGCCGTATTCATTGTC
Luciferase	ACGCTGAGTACTTCGAAATGTCC<br>TGCGAAATGCCCATACTGTTG
hF9 (F2/R2)	TTCACAAACTTCTAAGCTCACCCGTGCTG<br>AAAACAACCTGCCAAGGGAATTGACCTG
AAV ITR	GGAACCCCTAGTGATGGAGTT<br>CGGCCTCAGTGAGCGA
SpCas9	GCCTACCACGAGAAGTACCC<br>CTTGTCCACGTCGCTGTTGTC

Supplementary Table 3. Primers for T7E1 and off-target analysis.

Target site	Primer pair for genome PCR	
sgAlb target site	ACTTGCAAAGCCTAGAATCATGAAC	AGAGCAAAACCAGGGCTAACTCC
Off-target 1	GAGTGTGCTTTGTGCTAGCTG	CTTTACTTGTCTAGCCACCTCT
Off-target 2	GTTTGTTATAGGGTCTCTTCTCTCTAC	CTAGACTGCATCATTTCTACATTCATC
Off-target 3	TGAAAATTCTCCTTTGCCCTGAT	GTCTAGGAATTCCGCTGCAC
Off-target 4	CAGTACCCATGTGGATCGCC	GCCCTGTATGAAACTGGTGGT
Off-target 5	GAAATACTACAAAGTCCAACCCTTAAAC	CGTACAGAGTAAAGTTATAGCTGAGTTAG
Off-target 6	GTACAGCATGGCTTGCATCC	TCTAACTCTGCTATAATGTGCCTGA
Off-target 7	AGAGGGGCACATAGCAGGTA	CACATGGTTGGGTGTGCAAG
Off-target 8	TGTGACCAGGTTCTTGGAGC	ATTGTGACTGACTGGTCAATTCCT
Off-target 9	TATTGAATCAAGGTGACCCACAGA	TCACCAAAGCTAAATGCGATAGTT
Off-target 10	GTGCATAACGTAGGGCAGAGT	GCAAAGATTCCTTGGCCAAAAT
