# Supplementary material for: Low-dose AAV-CRISPR-mediated liver-specific knock-in restored hemostasis in neonatal hemophilia B mice with subtle antibody response
Source: Nat Commun. 2022 Nov 25;13:7275. doi: 10.1038/s41467-022-34898-y (PMC9700734; doi:10.1038/s41467-022-34898-y)
Supplement: Supplementary file 2 — Reporting Summary [file 41467_2022_34898_MOESM2_ESM.pdf]

nature portfolio

Corresponding author(s): Dr Bo FENG

Last updated by author(s): Jul 25, 2022

Reporting Summary

Nature Portfolio wishes to improve the reproducibility of the work that we publish. This form provides structure for consistency and transparency in reporting. For further information on Nature Portfolio policies, see our Editorial Policies and the Editorial Policy Checklist.

Statistics

For all statistical analyses, confirm that the following items are present in the figure legend, table legend, main text, or Methods section.

n/a	Confirmed	
☐	☒	The exact sample size (*n*) for each experimental group/condition, given as a discrete number and unit of measurement
☒	☐	A statement on whether measurements were taken from distinct samples or whether the same sample was measured repeatedly
☐	☒	The statistical test(s) used AND whether they are one- or two-sided<br>*Only common tests should be described solely by name; describe more complex techniques in the Methods section.*
☒	☐	A description of all covariates tested
☒	☐	A description of any assumptions or corrections, such as tests of normality and adjustment for multiple comparisons
☐	☒	A full description of the statistical parameters including central tendency (e.g. means) or other basic estimates (e.g. regression coefficient) AND variation (e.g. standard deviation) or associated estimates of uncertainty (e.g. confidence intervals)
☐	☒	For null hypothesis testing, the test statistic (e.g. *F*, *t*, *r*) with confidence intervals, effect sizes, degrees of freedom and *P* value noted<br>*Give P values as exact values whenever suitable.*
☒	☐	For Bayesian analysis, information on the choice of priors and Markov chain Monte Carlo settings
☒	☐	For hierarchical and complex designs, identification of the appropriate level for tests and full reporting of outcomes
☒	☐	Estimates of effect sizes (e.g. Cohen's *d*, Pearson's *r*), indicating how they were calculated

Our web collection on statistics for biologists contains articles on many of the points above.

Software and code

Policy information about availability of computer code

Data collection	No software was used for data collection
Data analysis	HISAT2 (2.2.1), HTSeq (v2.0), Samtools (1.9), R (4.2.0), python (3.9.5), RSEM (v1.0), IGV (v.2.11.4) was used for RNA-seq analysis.<br>Cas-OFFinder (v2.4), CCTop (v1.0), Synthego (v1.0) was used for off-target effect analysis.<br>ImageJ/Fiji v1.0 was used for PCR quantitative analysis.

For manuscripts utilizing custom algorithms or software that are central to the research but not yet described in published literature, software must be made available to editors and reviewers. We strongly encourage code deposition in a community repository (e.g. GitHub). See the Nature Portfolio guidelines for submitting code & software for further information.

Data

Policy information about availability of data

All manuscripts must include a data availability statement. This statement should provide the following information, where applicable:

- Accession codes, unique identifiers, or web links for publicly available datasets
- A description of any restrictions on data availability
- For clinical datasets or third party data, please ensure that the statement adheres to our policy

The authors confirm that all the data supporting the findings in this study are available within the article and its supplementary material. The RNA-seq data were

deposited into NCBI GEO database under accession code PRJNA782865 (https://www.ncbi.nlm.nih.gov/bioproject/PRJNA782865/) and the data sets are publicly released. The reference genome is mm10 and the annotation file (vM25) was downloaded from gencode.

Human research participants

Policy information about studies involving human research participants and Sex and Gender in Research.

Reporting on sex and gender	*Use the terms sex (biological attribute) and gender (shaped by social and cultural circumstances) carefully in order to avoid confusing both terms. Indicate if findings apply to only one sex or gender; describe whether sex and gender were considered in study design whether sex and/or gender was determined based on self-reporting or assigned and methods used. Provide in the source data disaggregated sex and gender data where this information has been collected, and consent has been obtained for sharing of individual-level data; provide overall numbers in this Reporting Summary. Please state if this information has not been collected. Report sex- and gender-based analyses where performed, justify reasons for lack of sex- and gender-based analysis.*
Population characteristics	*Describe the covariate-relevant population characteristics of the human research participants (e.g. age, genotypic information, past and current diagnosis and treatment categories). If you filled out the behavioural & social sciences study design questions and have nothing to add here, write "See above."*
Recruitment	*Describe how participants were recruited. Outline any potential self-selection bias or other biases that may be present and how these are likely to impact results.*
Ethics oversight	*Identify the organization(s) that approved the study protocol.*

Note that full information on the approval of the study protocol must also be provided in the manuscript.

Field-specific reporting

Please select the one below that is the best fit for your research. If you are not sure, read the appropriate sections before making your selection.

☒ Life sciences ☐ Behavioural & social sciences ☐ Ecological, evolutionary & environmental sciences

For a reference copy of the document with all sections, see nature.com/documents/nr-reporting-summary-flat.pdf

Life sciences study design

All studies must disclose on these points even when the disclosure is negative.

Sample size	All AAV knock-in experiments were performed with at least three biologically independent tests with negative controls. The efficiency measurements were conducted at least three independent replicates. The numbers of replicates was listed in the text and figure legends when applicable.
Data exclusions	Experimental handling may introduce variance. Occasional outliers were excluded.
Replication	The numbers of experimental replicates are listed and detailed in material and methods section, and in figure legends when applicable. All attempts at replication were successful.
Randomization	For all experiments, mice were treated with replicates and vehicle-only control were included to control for possible background.
Blinding	For all experiments in this project, the investigator were blinded to the group allocation during data collection and data analysis.

Reporting for specific materials, systems and methods

We require information from authors about some types of materials, experimental systems and methods used in many studies. Here, indicate whether each material, system or method listed is relevant to your study. If you are not sure if a list item applies to your research, read the appropriate section before selecting a response.

Materials & experimental systems

n/a	Involved in the study
☐	☒ Antibodies
☐	☒ Eukaryotic cell lines
☒	☐ Palaeontology and archaeology
☐	☒ Animals and other organisms
☒	☐ Clinical data
☒	☐ Dual use research of concern

Methods

n/a	Involved in the study
☒	☐ ChIP-seq
☒	☐ Flow cytometry
☒	☐ MRI-based neuroimaging

Antibodies

Antibodies used

Rabbit antibodies against human factor IX (Abcam, #ab97619)
Goat polyclonal anti-Albumin (Bethyl, #A90-134A)
Chicken anti-Rabbit IgG (H+L) Cross-Adsorbed Secondary Antibody, Alexa Fluor™ 594 (A-21442, Invitrogen)
Donkey anti-Goat IgG (H+L) Cross-Adsorbed Secondary Antibody, Alexa Fluor™ 488 (A-11055, Invitrogen)
Mouse monoclonal anti-Cas9 antibody (7A9-3A3) (sc-517386, Santa Cruz)
anti-mouse IgG conjugated with HRP (#7076S, Cell Signaling Technology)

Validation

1. https://www.abcam.com/products/primary-antibodies/factor-ixptc-antibody-ab97619.html
2. https://www.bioscience.co.uk/product~73015
3. https://www.scbt.com/p/cas9-antibody-7a9-3a3

Eukaryotic cell lines

Policy information about cell lines and Sex and Gender in Research

Cell line source(s)

293FT Cell - Thermo Fisher Scientific

Authentication

Purchased long time ago

Mycoplasma contamination

tested negative

Commonly misidentified lines
(See ICLAC register)

Name any commonly misidentified cell lines used in the study and provide a rationale for their use.

Animals and other research organisms

Policy information about studies involving animals; ARRIVE guidelines recommended for reporting animal research, and Sex and Gender in Research

Laboratory animals

Wild type ICR, C57BL/6, and mF9-/- (B6.129P2-F9tm1Dws/J) mice, at post natal day 1-3 (P1-P3), or at 6-12 weeks.

Wild animals

No wild animals were used in the study.

Reporting on sex

The experiments were performed on both males and females and findings were applicable to both males and females

Field-collected samples

No field collected samples were used in the study.

Ethics oversight

All experiments were approved by the Animal Experimentation Ethics Committee of the Chinese University of Hong Kong.

Note that full information on the approval of the study protocol must also be provided in the manuscript.
